# Supplementary material for: Stress-mediated convergence of splicing landscapes in male and female rock doves
Source: BMC Genomics. 2020 Mar 23;21:251. doi: 10.1186/s12864-020-6600-6 (PMC7092514; doi:10.1186/s12864-020-6600-6)
Supplement: Supplementary file 1 — Additional file 1: Table S1. Read counts and number of reads mapped for each sample. Table S2. Genome Annotation Statistics. Table S3. List of Spliced Genes. Table S4. Figure S1. Male vs. Female Biological Process GO Analysis. Figure S2. Male vs. Female Cellular Component GO Analysis. Figure S3. Male vs. Female Exon Motifs. Figure S4. Control vs. Stress Biological Process GO Analysis. Figure S5. Control vs. Stress Cellular Component GO Analysis. Figure S6. Control vs. Stress Exon Motifs. Figure S7. Distribution of Percent Spliced In (PSI) Values for the a) control vs stress analysis and also the b) male vs female analysis. [file 12864_2020_6600_MOESM1_ESM.docx]

**SUPPLEMENTARY TABLES & FIGURES**

Table S1. Read counts and number of reads mapped for each sample.

| Sample | Reads | Reads_Mapped |
| --- | --- | --- |
| blu-o-x-ATLAS_female_gonad_control | 13539296 | 7138070 |
| blu-o-x-ATLAS_female_hypothalamus_control | 3913288 | 1026493 |
| blu-o-x-ATLAS_female_pituitary_control | 18345014 | 9083093 |
| g-x-ATLAS_female_gonad_control | 16997078 | 8054619 |
| L-Blu13_male_gonad_control | 5054562 | 2148024 |
| L-Blu13_male_hypothalamus_control | 2543709 | 862955 |
| L-Blu13_male_pituitary_control | 9557351 | 2484308 |
| L-G107_male_gonad_control | 8031271 | 3340821 |
| L-G107_male_hypothalamus_control | 8024841 | 2239036 |
| L-G107_male_pituitary_control | 7287253 | 2131648 |
| L-G118_female_gonad_control | 18234825 | 7215637 |
| L-G118_female_hypothalamus_control | 3384528 | 1113945 |
| L-G118_female_pituitary_control | 6870567 | 3697542 |
| L-R3_male_gonad_control | 3156787 | 1270594 |
| L-R3_male_hypothalamus_control | 17241544 | 3365072 |
| L-R3_male_pituitary_control | 11940512 | 3320062 |
| L-R8_male_gonad_control | 20441428 | 8350547 |
| L-R8_male_hypothalamus_control | 709941 | 221369 |
| L-R8_male_pituitary_control | 10845174 | 3011774 |
| L-W33_male_gonad_control | 3975356 | 1608117 |
| L-W33_male_hypothalamus_control | 7165380 | 2549508 |
| L-W33_male_pituitary_control | 14252346 | 3921480 |
| L-W3_male_gonad_control | 3485822 | 1366433 |
| L-W3_male_hypothalamus_control | 890990 | 242947 |
| L-W3_male_pituitary_control | 10232066 | 3465157 |
| L-W4_male_gonad_control | 4369194 | 1706317 |
| L-W4_male_hypothalamus_control | 11513277 | 4224149 |
| L-W4_male_pituitary_control | 10561933 | 3107349 |
| r6-x_female_gonad_control | 13911152 | 7755998 |
| r6-x_female_hypothalamus_control | 15717373 | 7378104 |
| r6-x_female_pituitary_control | 5698441 | 2551178 |
| R-G106_female_gonad_control | 22437804 | 9873172 |
| R-G106_female_hypothalamus_control | 20496509 | 7267199 |
| R-G106_female_pituitary_control | 9801969 | 2524604 |
| R-R20_female_gonad_control | 11783739 | 5070042 |
| R-R20_female_hypothalamus_control | 9636064 | 2261311 |
| R-R20_female_pituitary_control | 9117568 | 2806792 |
| R-R9_female_gonad_control | 7157493 | 3184249 |
| R-R9_female_hypothalamus_control | 23579036 | 7480922 |
| R-R9_female_pituitary_control | 13085336 | 3811543 |
| r-r-x-ATLAS_female_gonad_control | 17979019 | 8958258 |
| r-r-x-ATLAS_female_hypothalamus_control | 7739732 | 2289400 |
| r-r-x-ATLAS_female_pituitary_control | 4440924 | 1623597 |
| r-r-x-ATLAS-R2XR_female_gonad_control | 5868581 | 2903751 |
| r-r-x-ATLAS-R2XR_female_hypothalamus_control | 7739732 | 2289098 |
| r-r-x-ATLAS-R2XR_female_pituitary_control | 10597141 | 4582916 |
| R-W44_female_gonad_control | 28396609 | 11572936 |
| R-W44_female_hypothalamus_control | 30086026 | 8598958 |
| R-W44_female_pituitary_control | 7757736 | 1961997 |
| R-Y108-W29_male_gonad_control | 13166331 | 5340667 |
| R-Y108-W29_male_hypothalamus_control | 3520956 | 1147454 |
| R-Y108-W29_male_pituitary_control | 17123184 | 3869459 |
| s-x-ATLAS_female_gonad_control | 14518773 | 5898429 |
| s-x-ATLAS_female_hypothalamus_control | 14886148 | 3759606 |
| s-x-ATLAS_female_pituitary_control | 3483526 | 1435392 |
| w191-r1_female_gonad_control | 6115725 | 3262296 |
| w191-r1_female_hypothalamus_control | 6947942 | 3059278 |
| w191-r1_female_pituitary_control | 6322612 | 2919302 |
| x-blk-blk-ATLAS_male_gonad_control | 14105178 | 6211153 |
| x-blk-blk-ATLAS_male_hypothalamus_control | 11947014 | 4031545 |
| x-blk-blk-ATLAS_male_pituitary_control | 3168840 | 1299083 |
| x-blu-o-ATLAS_male_pituitary_control | 16794696 | 5979554 |
| x-g-ATLAS_male_gonad_control | 3219853 | 1241673 |
| x-g-ATLAS_male_hypothalamus_control | 10344992 | 3329742 |
| x-g-ATLAS_male_pituitary_control | 12723547 | 4990470 |
| x-g-g-ATLAS_female_gonad_control | 16207421 | 8380847 |
| x-g-g-ATLAS_male_gonad_control | 13654678 | 5968115 |
| x-g-g-ATLAS_male_hypothalamus_control | 9297715 | 2167600 |
| x-g-g-ATLAS_male_pituitary_control | 11866001 | 3447849 |
| x-g-g-g-ATLAS_male_gonad_control | 13304832 | 5809012 |
| x-g-g-g-ATLAS_male_pituitary_control | 10084225 | 4345194 |
| x-y-s-ATLAS_male_gonad_control | 11852142 | 5076732 |
| x-y-s-ATLAS_male_pituitary_control | 4123699 | 1731920 |
| blu7-x_female_gonad_stress | 8094547 | 4113564 |
| blu7-x_female_hypothalamus_stress | 7593322 | 3109295 |
| blu7-x_female_pituitary_stress | 7513155 | 3610239 |
| g101-x_female_gonad_stress | 6407199 | 2873211 |
| g101-x_female_hypothalamus_stress | 6254427 | 2912340 |
| g101-x_female_pituitary_stress | 6728144 | 3145116 |
| g105-x_male_gonad_stress | 7306761 | 3075298 |
| g105-x_male_hypothalamus_stress | 6673149 | 2822830 |
| g105-x_male_pituitary_stress | 6425135 | 2633392 |
| L-Blu10-W37_female_gonad_stress | 17469609 | 6850624 |
| L-Blu10-W37_female_hypothalamus_stress | 1179024 | 324056 |
| L-Blu10-W37_female_pituitary_stress | 9527432 | 2691882 |
| L-Blu123-W38_male_gonad_stress | 1659349 | 567266 |
| L-Blu123-W38_male_hypothalamus_stress | 2423067 | 762181 |
| L-Blu123-W38_male_pituitary_stress | 6922829 | 1890694 |
| L-O116_male_gonad_stress | 3199634 | 1318900 |
| L-O116_male_hypothalamus_stress | 13912972 | 3448134 |
| L-O116_male_pituitary_stress | 19798845 | 6360620 |
| L-O123-W28_male_gonad_stress | 2149496 | 834525 |
| L-O123-W28_male_hypothalamus_stress | 3557306 | 1087775 |
| L-O123-W28_male_pituitary_stress | 4751362 | 1533019 |
| L-R2_male_gonad_stress | 16807754 | 6841724 |
| L-R2_male_hypothalamus_stress | 22076763 | 7725812 |
| L-R2_male_pituitary_stress | 2810140 | 625899 |
| L-Y101_male_gonad_stress | 308816 | 115779 |
| L-Y101_male_hypothalamus_stress | 70251163 | 14407783 |
| L-Y101_male_pituitary_stress | 19136564 | 6574864 |
| L-Y111_male_gonad_stress | 9230118 | 3512151 |
| L-Y111_male_hypothalamus_stress | 9894568 | 2519087 |
| L-Y111_male_pituitary_stress | 17660375 | 7564359 |
| o122-g2-x_male_gonad_stress | 7957606 | 3384679 |
| o122-g2-x_male_hypothalamus_stress | 5366274 | 2058503 |
| o122-g2-x_male_pituitary_stress | 6887620 | 3039002 |
| o124-y124-x_male_gonad_stress | 5734998 | 2617034 |
| o124-y124-x_male_hypothalamus_stress | 6789454 | 2255470 |
| o124-y124-x_male_pituitary_stress | 5017287 | 2291771 |
| R-Blu12_female_gonad_stress | 22935635 | 11142871 |
| R-Blu12_female_hypothalamus_stress | 20015443 | 6687745 |
| R-Blu12_female_pituitary_stress | 12523257 | 3159854 |
| R-Blu8-W30_male_gonad_stress | 738184 | 272217 |
| R-Blu8-W30_male_hypothalamus_stress | 872265 | 214030 |
| R-Blu8-W30_male_pituitary_stress | 10319307 | 2951617 |
| R-G124_male_gonad_stress | 8065220 | 3091398 |
| R-G124_male_hypothalamus_stress | 5549401 | 1931536 |
| R-G124_male_pituitary_stress | 10373853 | 2838182 |
| R-O106-W27_male_gonad_stress | 13040039 | 5420405 |
| R-O106-W27_male_hypothalamus_stress | 8026760 | 2275720 |
| R-O106-W27_male_pituitary_stress | 9039979 | 2273709 |
| R-W7_female_gonad_stress | 38828116 | 17987395 |
| R-W7_female_hypothalamus_stress | 14314986 | 3502430 |
| R-W7_female_pituitary_stress | 5087929 | 1455479 |
| R-Y116_female_gonad_stress | 19188040 | 7901456 |
| R-Y116_female_hypothalamus_stress | 10245357 | 2139400 |
| R-Y116_female_pituitary_stress | 15936063 | 5914010 |
| x-g113_female_gonad_stress | 8955491 | 3807474 |
| x-g113_female_hypothalamus_stress | 9030955 | 2204257 |
| x-g113_female_pituitary_stress | 6619134 | 3087377 |
| x-r23_female_gonad_stress | 7531666 | 3037465 |
| x-r23_female_hypothalamus_stress | 6394384 | 2817517 |
| x-r23_female_pituitary_stress | 8287246 | 3912273 |
| x-w24-w49_female_gonad_stress | 7594432 | 3760209 |
| x-w24-w49_female_hypothalamus_stress | 8436279 | 3419628 |
| x-w24-w49_female_pituitary_stress | 8721184 | 4165458 |
| x-w31_female_gonad_stress | 8607761 | 3799892 |
| x-w31_female_hypothalamus_stress | 7054101 | 2450528 |
| x-w31_female_pituitary_stress | 8109160 | 3598354 |
| x-y106_female_gonad_stress | 6952070 | 3175822 |
| x-y106_female_hypothalamus_stress | 8289761 | 3451235 |
| x-y106_female_pituitary_stress | 8019660 | 3609927 |
| y104-w190_female_gonad_stress | 6319380 | 3197575 |
| y104-w190_female_hypothalamus_stress | 6298066 | 2769875 |
| y104-w190_female_pituitary_stress | 5477743 | 2618946 |
| y110-x_male_gonad_stress | 5241570 | 2265136 |
| y110-x_male_hypothalamus_stress | 8365538 | 3526141 |
| y110-x_male_pituitary_stress | 4722833 | 2257143 |

Table S2. Genome Annotation Statistics. Provided in this table are the statistics for the annotation of the chromosome-level assembly ([GCA_001887795.1](https://www.ncbi.nlm.nih.gov/assembly/GCA_001887795.1)). Value column contains lengths/numbers/counts denoted by the statistic indicated in the first column.

| Statistic | Value |
| --- | --- |
| Total sequence length | 1018016946 |
| Number of genes | 14463 |
| Number of mRNAs | 33890 |
| Number of exons | 449071 |
| Number of introns | 415181 |
| Number of CDS | 33890 |
| Overlapping genes | 1523 |
| Contained genes | 143 |
| Total gene length | 342131760 |
| Total mRNA length | 954855887 |
| Total exon length | 119038219 |
| Total intron length | 836648030 |
| Total CDS length | 61662777 |
| Shortest gene length | 144 |
| Shortest mRNA length | 144 |
| Shortest exon length | 1 |
| Shortest intron length | 20 |
| Shortest CDS length | 21 |
| Longest gene length | 342216 |
| Longest mRNA length | 310290 |
| Longest exon length | 15288 |
| Longest intron length | 49478 |
| Longest CDS length | 35607 |
| mean gene length | 23656 |
| mean mRNA length | 28175 |
| mean exon length | 265 |
| mean intron length | 2015 |
| mean CDS length | 1819 |
| % of genome covered by genes | 33.6 |
| % of genome covered by CDS | 6.1 |
| mean number of mRNAs per gene | 2 |
| mean number of exons per mRNA | 13 |
| mean number of introns per mRNA | 12 |

Table S3. List of Spliced Genes. This list contains the Entrez ID for all genes in which an alternative splicing event was found during this study. Column headers refer to the comparison (M= male, F= female, H= hypothalamus, P= pituitary, G= gonad, C= control, S= stress). This table is a compiled version of Table S4, which contains all splicing event information.

| Spliced Genes | | | | | | | | | |
| --- | --- | --- | --- | --- | --- | --- | --- | --- | --- |
| Male - Female Alt. Splicing | | | | Control - Stress Alt. Splicing | | | | | |
| MHC-FHC | MHS-FHS | MPC-FPC | MPS-FPS | MHC-MHS | FHC-FHS | MPC-MPS | FPC-FPS | MGC-MGS | FGC-FGS |
| 102083532 | 102083948 | 102083465 | 102083465 | 102084143 | 102083434 | 102083892 | 102083807 | 102040246 | 102083405 |
| 102083613 | 102084196 | 102084030 | 102083544 | 102084328 | 102083979 | 102084037 | 102083833 | 102083695 | 102083474 |
| 102083933 | 102084230 | 102084176 | 102083569 | 102084570 | 102084230 | 102084176 | 102083922 | 102084031 | 102083685 |
| 102083979 | 102084238 | 102084408 | 102083595 | 102084643 | 102084352 | 102084196 | 102083948 | 102084074 | 102083945 |
| 102084238 | 102084706 | 102084611 | 102083833 | 102084933 | 102084764 | 102084780 | 102084088 | 102084328 | 102084037 |
| 102084352 | 102085081 | 102084780 | 102083922 | 102084977 | 102084935 | 102084933 | 102084143 | 102085052 | 102084327 |
| 102084570 | 102085598 | 102084901 | 102084053 | 102085148 | 102085198 | 102085253 | 102084175 | 102086211 | 102084977 |
| 102084643 | 102085623 | 102085525 | 102084155 | 102085302 | 102085412 | 102085426 | 102084238 | 102086283 | 102085385 |
| 102085132 | 102085663 | 102085547 | 102084175 | 102085483 | 102085568 | 102085547 | 102084369 | 102086997 | 102085587 |
| 102085412 | 102086227 | 102085568 | 102084295 | 102085568 | 102085607 | 102085568 | 102084408 | 102087010 | 102085728 |
| 102085483 | 102086573 | 102085569 | 102084570 | 102085700 | 102085663 | 102085671 | 102084570 | 102088507 | 102085809 |
| 102085598 | 102087151 | 102085587 | 102084695 | 102085923 | 102087151 | 102085770 | 102085103 | 102089105 | 102086362 |
| 102085700 | 102087631 | 102085809 | 102084802 | 102086113 | 102087735 | 102085811 | 102085385 | 102090542 | 102086384 |
| 102085822 | 102088288 | 102085974 | 102085426 | 102086160 | 102088300 | 102086063 | 102085809 | 102090846 | 102086750 |
| 102085942 | 102088300 | 102086187 | 102085500 | 102086227 | 102088706 | 102086718 | 102085836 | 102091285 | 102087294 |
| 102085974 | 102088757 | 102086210 | 102085663 | 102086287 | 102088967 | 102086865 | 102086043 | 102091335 | 102087340 |
| 102086160 | 102088785 | 102086221 | 102085809 | 102086537 | 102089203 | 102087010 | 102086708 | 102092374 | 102088149 |
| 102086227 | 102088881 | 102087186 | 102085833 | 102087006 | 102089321 | 102087178 | 102086868 | 102092436 | 102088253 |
| 102086328 | 102088892 | 102087259 | 102085982 | 102087276 | 102089730 | 102087434 | 102087048 | 102092590 | 102088659 |
| 102086358 | 102090369 | 102087423 | 102086043 | 102087608 | 102089918 | 102088097 | 102087100 | 102092763 | 102088826 |
| 102086394 | 102090470 | 102087434 | 102086064 | 102087749 | 102090083 | 102088373 | 102087352 | 102093249 | 102089159 |
| 102086537 | 102090698 | 102087986 | 102086217 | 102088088 | 102090599 | 102088507 | 102087515 | 102093887 | 102089395 |
| 102086804 | 102090771 | 102088097 | 102086396 | 102088201 | 102090771 | 102088697 | 102088066 | 102093951 | 102090176 |
| 102087006 | 102091175 | 102088149 | 102086573 | 102088300 | 102091346 | 102088721 | 102088149 | 102094063 | 102090757 |
| 102087405 | 102091392 | 102088241 | 102086811 | 102088390 | 102091571 | 102088726 | 102088178 | 102095142 | 102090762 |
| 102087608 | 102091530 | 102088438 | 102086868 | 102088688 | 102092189 | 102089159 | 102088182 | 102095854 | 102090828 |
| 102088057 | 102092123 | 102088507 | 102087294 | 102088881 | 102093082 | 102089353 | 102088368 | 102096684 | 102090939 |
| 102088088 | 102092393 | 102088530 | 102087407 | 102089252 | 102093197 | 102089916 | 102088530 | 102096732 | 102091285 |
| 102088314 | 102092428 | 102088599 | 102087410 | 102089640 | 102093485 | 102090035 | 102088550 | 102097369 | 102091510 |
| 102088390 | 102092530 | 102088697 | 102087669 | 102089642 | 102093951 | 102090452 | 102088697 | 102097377 | 102091597 |
| 102088757 | 102093831 | 102088856 | 102088013 | 102089699 | 102094305 | 102090756 | 102088929 | 102097852 | 102091752 |
| 102089233 | 102094305 | 102088965 | 102088697 | 102089841 | 102095309 | 102090828 | 102089128 | 102098881 | 102092251 |
| 102089252 | 102094490 | 102089159 | 102088856 | 102090171 | 102095597 | 102091119 | 102089159 | 102098904 | 102093022 |
| 102089640 | 102095309 | 102089384 | 102089104 | 102090174 | 102096323 | 102091392 | 102089352 | 104034471 | 102093169 |
| 102089642 | 102095462 | 102089442 | 102089528 | 102090337 | 102096431 | 102091459 | 102090239 | 104914348 | 102093450 |
| 102089681 | 102096169 | 102089513 | 102090315 | 102090375 | 102096569 | 102091538 | 102090337 | 106146053 | 102093782 |
| 102089699 | 102096295 | 102089575 | 102090423 | 102090599 | 102097170 | 102091932 | 102090392 | 110355067 | 102094364 |
| 102089748 | 102096327 | 102089633 | 102090586 | 102090698 | 102097866 | 102092000 | 102090571 | 110358081 | 102094387 |
| 102090171 | 102096698 | 102089772 | 102090591 | 102090947 | 102098594 | 102092721 | 102090591 | 110360169 | 102094439 |
| 102090337 | 102096853 | 102090098 | 102090657 | 102091278 | 102098752 | 102093071 | 102091060 | 110364063 | 102094490 |
| 102090375 | 102097170 | 102090171 | 102090682 | 102091397 | 102098932 | 102093169 | 102091116 | 110365763 | 102094693 |
| 102090408 | 102097217 | 102090335 | 102090822 | 102091457 | 103915628 | 102093556 | 102091355 |  | 102095251 |
| 102091116 | 102097356 | 102090397 | 102091116 | 102091617 | 104521661 | 102093886 | 102091392 |  | 102095621 |
| 102091304 | 102097778 | 102090423 | 102091346 | 102091635 | 106146132 | 102094617 | 102091432 |  | 102096077 |
| 102091397 | 102097815 | 102090452 | 102091392 | 102091885 | 110356509 | 102094712 | 102091635 |  | 102096100 |
| 102091617 | 102098187 | 102090468 | 102091432 | 102091929 |  | 102095309 | 102091925 |  | 102096300 |
| 102091635 | 102098932 | 102090563 | 102091538 | 102092123 |  | 102095440 | 102092349 |  | 102096391 |
| 102091885 | 103901448 | 102090571 | 102091925 | 102092245 |  | 102095514 | 102092361 |  | 102096453 |
| 102092000 | 104833389 | 102090702 | 102092265 | 102092335 |  | 102096292 | 102092413 |  | 102097035 |
| 102092021 |  | 102090819 | 102092269 | 102092451 |  | 102096408 | 102092628 |  | 102097058 |
| 102092093 |  | 102091060 | 102092453 | 102093138 |  | 102096431 | 102093135 |  | 102097073 |
| 102092123 |  | 102091177 | 102092603 | 102093302 |  | 102096569 | 102093178 |  | 102097185 |
| 102092335 |  | 102091538 | 102092628 | 102093328 |  | 102096686 | 102093545 |  | 102097544 |
| 102092554 |  | 102091563 | 102093135 | 102093658 |  | 102097088 | 102093700 |  | 102097709 |
| 102092636 |  | 102091635 | 102093180 | 102094350 |  | 102097170 | 102093886 |  | 102097849 |
| 102093302 |  | 102091662 | 102093744 | 102094490 |  | 102097480 | 102094060 |  | 102097852 |
| 102093328 |  | 102091934 | 102093822 | 102094943 |  | 102097560 | 102094099 |  | 102098006 |
| 102093658 |  | 102092371 | 102093862 | 102095370 |  | 102097670 | 102094379 |  | 102098396 |
| 102094122 |  | 102092413 | 102093886 | 102095379 |  | 102097851 | 102094555 |  | 102098544 |
| 102094263 |  | 102093169 | 102094122 | 102095769 |  | 102098015 | 102094726 |  | 105399520 |
| 102094350 |  | 102093328 | 102094290 | 102095997 |  | 102098180 | 102095052 |  | 105414115 |
| 102094422 |  | 102093700 | 102095946 | 102096692 |  | 102098190 | 102095188 |  | 106146053 |
| 102094674 |  | 102093744 | 102096491 | 102096928 |  | 102098326 | 102095228 |  | 106885273 |
| 102094712 |  | 102093801 | 102096700 | 102097377 |  | 102098553 | 102095595 |  | 110355089 |
| 102094726 |  | 102094310 | 102096733 | 102097419 |  | 102098732 | 102095706 |  | 110355790 |
| 102094838 |  | 102094490 | 102096825 | 102097571 |  | 102098891 | 102095733 |  | 110355889 |
| 102095024 |  | 102094583 | 102097185 | 102097653 |  | 103533213 | 102095735 |  | 110358344 |
| 102095747 |  | 102094693 | 102097377 | 102098180 |  | 103896199 | 102095946 |  | 110361585 |
| 102095782 |  | 102094712 | 102097435 | 102098416 |  | 106145708 | 102096498 |  | 110363298 |
| 102095911 |  | 102095199 | 102097572 | 102098594 |  |  | 102096541 |  | 110365053 |
| 102095997 |  | 102095309 | 102097709 | 103895355 |  |  | 102096550 |  |  |
| 102096169 |  | 102095595 | 102097851 | 106145708 |  |  | 102096691 |  |  |
| 102096298 |  | 102095850 | 102098182 | 106145849 |  |  | 102096700 |  |  |
| 102096825 |  | 102096025 | 102098203 | 106145910 |  |  | 102096749 |  |  |
| 102097097 |  | 102096292 | 102098275 | 110362363 |  |  | 102096825 |  |  |
| 102097377 |  | 102096298 | 102098594 |  |  |  | 102097332 |  |  |
| 102097419 |  | 102096534 | 102098693 |  |  |  | 102097377 |  |  |
| 102097653 |  | 102096543 | 102098898 |  |  |  | 102097566 |  |  |
| 102097687 |  | 102096569 | 103901317 |  |  |  | 102097661 |  |  |
| 102098416 |  | 102096686 | 104022714 |  |  |  | 102097848 |  |  |
| 102098594 |  | 102096698 | 105403301 |  |  |  | 102098083 |  |  |
| 106145910 |  | 102096732 | 105411610 |  |  |  | 102098089 |  |  |
| 106145966 |  | 102096781 | 106145760 |  |  |  | 102098180 |  |  |
| 110358848 |  | 102097223 | 106145801 |  |  |  | 102098275 |  |  |
| 110365212 |  | 102097377 | 106146053 |  |  |  | 102098756 |  |  |
|  |  | 102097566 | 106885273 |  |  |  | 102098898 |  |  |
|  |  | 102097634 | 106891296 |  |  |  | 103912455 |  |  |
|  |  | 102097851 | 110357300 |  |  |  | 104022714 |  |  |
|  |  | 102097908 | 110357802 |  |  |  | 105401876 |  |  |
|  |  | 102097941 | 110363815 |  |  |  | 105408022 |  |  |
|  |  | 102098436 | 110365053 |  |  |  | 105411610 |  |  |
|  |  | 102098693 |  |  |  |  | 106145708 |  |  |
|  |  | 102098765 |  |  |  |  | 110355187 |  |  |
|  |  | 102098814 |  |  |  |  | 110356341 |  |  |
|  |  | 104836833 |  |  |  |  |  |  |  |
|  |  | 106145967 |  |  |  |  |  |  |  |
|  |  | 110359246 |  |  |  |  |  |  |  |
|  |  | 110362309 |  |  |  |  |  |  |  |
|  |  | 110365155 |  |  |  |  |  |  |  |

Table S4. Table of all statistically significant events. This table depicts, for each treatment comparison (First column), the gene wherein the splicing occurred (2^nd^ column), the genomic location (3^rd^ column), event type (4^th^ column), delta PSI (5^th^ column), and event probability (6^th^ column).

| Comparison | GeneID | Location | EventType | deltaPSI | Probability |
| --- | --- | --- | --- | --- | --- |
| female_gonad_control vs female_gonad_stress | 102094439 | CM007525.1:21983297-21983299 | AA | 0.44564 | 0.98 |
| female_gonad_control vs female_gonad_stress | 102086362 | CM007525.1:11968646-11968660 | AA | 0.55932 | 0.996 |
| female_gonad_control vs female_gonad_stress | 102090757 | CM007525.1:106957102-106957172 | CE | -0.49142 | 0.983 |
| female_gonad_control vs female_gonad_stress | 102098006 | CM007525.1:73793765-73796513 | AF | -0.49836 | 0.981 |
| female_gonad_control vs female_gonad_stress | 102085809 | CM007525.1:135001346-135001420 | CE | 0.43078 | 0.975 |
| female_gonad_control vs female_gonad_stress | 102085809 | CM007525.1:134996935-134997000 | CE | 0.59119 | 0.997 |
| female_gonad_control vs female_gonad_stress | 102085809 | CM007525.1:134993920-134993994 | CE | 0.46049 | 0.967 |
| female_gonad_control vs female_gonad_stress | 102094693 | CM007525.1:192535192-192535251 | CE | -0.2673 | 0.95 |
| female_gonad_control vs female_gonad_stress | 106885273 | CM007525.1:69853414-69853440 | CE | 0.47598 | 0.989 |
| female_gonad_control vs female_gonad_stress | 102089395 | CM007534.1:17328736-17328831 | CE | -0.24025 | 0.958 |
| female_gonad_control vs female_gonad_stress | 102097058 | CM007534.1:16714312-16715026 | AA | -0.24296 | 0.98 |
| female_gonad_control vs female_gonad_stress | 102088253 | CM007535.1:5703255-5703277 | AA | -0.47776 | 0.987 |
| female_gonad_control vs female_gonad_stress | 102097709 | CM007536.1:15598309-15598390 | RI | 0.53175 | 0.997 |
| female_gonad_control vs female_gonad_stress | 102090762 | CM007537.1:13876560-13876681 | CE | -0.26076 | 0.956 |
| female_gonad_control vs female_gonad_stress | 102084977 | CM007538.1:4221391-4221635 | RI | 0.51051 | 0.987 |
| female_gonad_control vs female_gonad_stress | 102087340 | CM007538.1:2276140-2276175 | AD | 0.67414 | 1.0 |
| female_gonad_control vs female_gonad_stress | 110358344 | CM007539.1:7980849-7981022 | CE | 0.40971 | 0.968 |
| female_gonad_control vs female_gonad_stress | 102083945 | CM007539.1:4246897-4246957 | CE | -0.5084 | 0.997 |
| female_gonad_control vs female_gonad_stress | 102084327 | CM007540.1:2312174-2312233 | CE | -0.50139 | 0.969 |
| female_gonad_control vs female_gonad_stress | 106146053 | CM007540.1:2231970-2232190 | AA | 0.33137 | 0.957 |
| female_gonad_control vs female_gonad_stress | 102096391 | CM007542.1:2881414-2881507 | CE | 0.44924 | 0.997 |
| female_gonad_control vs female_gonad_stress | 102095621 | CM007542.1:4408278-4408366 | CE | -0.41354 | 0.989 |
| female_gonad_control vs female_gonad_stress | 102097073 | CM007526.1:111927763-111928007 | CE | -0.13815 | 0.972 |
| female_gonad_control vs female_gonad_stress | 102097073 | CM007526.1:111925747-111925877 | CE | -0.14273 | 0.954 |
| female_gonad_control vs female_gonad_stress | 102097849 | CM007526.1:82568291-82568329 | CE | 0.40732 | 0.963 |
| female_gonad_control vs female_gonad_stress | 110365053 | CM007526.1:134453095-134453198 | CE | 0.40951 | 0.97 |
| female_gonad_control vs female_gonad_stress | 102094364 | CM007526.1:100917258-100918522 | TE | -0.52408 | 0.997 |
| female_gonad_control vs female_gonad_stress | 102094364 | CM007526.1:100921212-100921375 | TE | 0.52199 | 0.992 |
| female_gonad_control vs female_gonad_stress | 110355889 | CM007543.1:1895623-1895769 | CE | -0.10005 | 0.95 |
| female_gonad_control vs female_gonad_stress | 110355889 | CM007543.1:1894394-1894454 | CE | -0.2321 | 0.985 |
| female_gonad_control vs female_gonad_stress | 102086750 | CM007543.1:38978-39182 | CE | 0.33861 | 0.955 |
| female_gonad_control vs female_gonad_stress | 102088659 | CM007544.1:15148-15171 | AD | 0.51481 | 0.981 |
| female_gonad_control vs female_gonad_stress | 110355790 | CM007544.1:626859-626936 | CE | 0.30645 | 0.952 |
| female_gonad_control vs female_gonad_stress | 102088149 | CM007546.1:2919903-2920073 | CE | -0.3108 | 0.957 |
| female_gonad_control vs female_gonad_stress | 102094387 | CM007550.1:2970927-2970978 | AA | -0.44701 | 0.98 |
| female_gonad_control vs female_gonad_stress | 102092251 | CM007550.1:3233310-3233466 | CE | -0.12327 | 0.979 |
| female_gonad_control vs female_gonad_stress | 102092251 | CM007550.1:3232999-3233160 | CE | -0.14301 | 0.991 |
| female_gonad_control vs female_gonad_stress | 102092251 | CM007550.1:3232422-3232642 | CE | -0.10796 | 0.976 |
| female_gonad_control vs female_gonad_stress | 102092251 | CM007550.1:3232199-3232239 | CE | -0.11938 | 0.972 |
| female_gonad_control vs female_gonad_stress | 105414115 | CM007551.1:1482866-1483108 | CE | -0.54238 | 0.983 |
| female_gonad_control vs female_gonad_stress | 102087294 | CM007527.1:15416415-15416486 | CE | 0.76484 | 1.0 |
| female_gonad_control vs female_gonad_stress | 102084037 | CM007527.1:3683052-3683278 | RI | -0.78956 | 1.0 |
| female_gonad_control vs female_gonad_stress | 102084037 | CM007527.1:3683031-3683051 | CE | -0.76758 | 1.0 |
| female_gonad_control vs female_gonad_stress | 102084037 | CM007527.1:3682980-3683030 | AD | -0.71322 | 1.0 |
| female_gonad_control vs female_gonad_stress | 102084037 | CM007527.1:3682882-3682895 | AA | -0.66068 | 1.0 |
| female_gonad_control vs female_gonad_stress | 102083405 | CM007528.1:46297-46308 | AD | 0.59134 | 0.984 |
| female_gonad_control vs female_gonad_stress | 102093169 | CM007528.1:12058160-12059598 | RI | 0.3009 | 0.981 |
| female_gonad_control vs female_gonad_stress | 102093169 | CM007528.1:12059599-12059614 | CE | 0.29288 | 0.983 |
| female_gonad_control vs female_gonad_stress | 102098396 | CM007528.1:37650177-37650215 | AA | 0.39593 | 0.95 |
| female_gonad_control vs female_gonad_stress | 102091597 | CM007528.1:51113783-51113804 | CE | 0.37317 | 0.973 |
| female_gonad_control vs female_gonad_stress | 102097852 | CM007523.1:374355-374441 | CE | 0.38736 | 0.981 |
| female_gonad_control vs female_gonad_stress | 102093782 | CM007529.1:16043526-16043698 | AA | 0.71435 | 0.999 |
| female_gonad_control vs female_gonad_stress | 102090939 | CM007529.1:42984153-42984487 | RI | -0.37993 | 0.972 |
| female_gonad_control vs female_gonad_stress | 102086384 | CM007529.1:6375719-6375722 | AA | -0.76876 | 1.0 |
| female_gonad_control vs female_gonad_stress | 102085728 | CM007530.1:19865214-19865395 | CE | -0.38642 | 0.993 |
| female_gonad_control vs female_gonad_stress | 102088826 | CM007530.1:28764459-28764537 | CE | 0.36933 | 0.958 |
| female_gonad_control vs female_gonad_stress | 102089159 | CM007530.1:34836488-34836592 | CE | 0.48107 | 0.996 |
| female_gonad_control vs female_gonad_stress | 102089159 | CM007530.1:34830759-34830863 | CE | -0.4489 | 0.978 |
| female_gonad_control vs female_gonad_stress | 102091510 | CM007531.1:547662-548098 | AA | 0.33569 | 0.959 |
| female_gonad_control vs female_gonad_stress | 102085385 | CM007531.1:14765898-14765946 | CE | 0.41312 | 0.97 |
| female_gonad_control vs female_gonad_stress | 102098544 | CM007531.1:4610829-4610844 | CE | -0.61512 | 0.998 |
| female_gonad_control vs female_gonad_stress | 102097544 | CM007531.1:5658914-5659092 | TE | -0.52091 | 0.993 |
| female_gonad_control vs female_gonad_stress | 102097544 | CM007531.1:5659092-5659092 | TE | 0.52504 | 0.997 |
| female_gonad_control vs female_gonad_stress | 110363298 | CM007532.1:18086112-18086280 | CE | 0.48439 | 0.979 |
| female_gonad_control vs female_gonad_stress | 102095251 | CM007532.1:23588403-23588538 | CE | 0.40802 | 0.959 |
| female_gonad_control vs female_gonad_stress | 102095251 | CM007532.1:23591215-23591332 | CE | 0.4123 | 0.987 |
| female_gonad_control vs female_gonad_stress | 105399520 | CM007532.1:26432746-26432746 | TS | -0.48822 | 0.985 |
| female_gonad_control vs female_gonad_stress | 105399520 | CM007532.1:26432592-26432746 | TS | 0.48475 | 0.986 |
| female_gonad_control vs female_gonad_stress | 102096077 | CM007532.1:3415711-3415726 | AA | -0.37623 | 0.995 |
| female_gonad_control vs female_gonad_stress | 102096100 | CM007533.1:4118963-4118963 | AD | -0.38174 | 0.968 |
| female_gonad_control vs female_gonad_stress | 102083474 | CM007533.1:19308540-19308678 | AD | -0.41479 | 0.968 |
| female_gonad_control vs female_gonad_stress | 102090828 | CM007533.1:21310068-21310220 | TS | 0.4808 | 0.986 |
| female_gonad_control vs female_gonad_stress | 102090828 | CM007533.1:21294695-21294785 | TS | -0.51152 | 0.995 |
| female_gonad_control vs female_gonad_stress | 102091285 | CM007533.1:7580614-7580818 | CE | -0.13149 | 0.968 |
| female_gonad_control vs female_gonad_stress | 102085587 | CM007524.1:25512484-25512510 | AD | -0.44319 | 0.972 |
| female_gonad_control vs female_gonad_stress | 102093450 | CM007524.1:4688695-4688749 | AA | 0.3118 | 0.972 |
| female_gonad_control vs female_gonad_stress | 110355089 | KV877987.1:1561506-1561531 | AD | 0.61961 | 0.991 |
| female_gonad_control vs female_gonad_stress | 102096453 | KV877988.1:1927985-1928086 | AA | -0.6101 | 0.976 |
| female_gonad_control vs female_gonad_stress | 102083685 | KV877988.1:5150255-5150489 | CE | -0.38951 | 0.979 |
| female_gonad_control vs female_gonad_stress | 102096300 | KV877999.1:10697634-10697654 | AD | 0.19804 | 0.957 |
| female_gonad_control vs female_gonad_stress | 102093022 | KV878001.1:3426073-3426202 | CE | 0.36846 | 0.964 |
| female_gonad_control vs female_gonad_stress | 102094490 | KV878004.1:3082306-3082345 | AD | -0.52215 | 0.961 |
| female_gonad_control vs female_gonad_stress | 102094490 | KV878004.1:3086890-3086991 | AA | -0.357 | 0.952 |
| female_gonad_control vs female_gonad_stress | 102097185 | KV878008.1:1518012-1518726 | CE | -0.40606 | 0.963 |
| female_gonad_control vs female_gonad_stress | 102090176 | KV878011.1:2505831-2505873 | AA | -0.58677 | 0.993 |
| female_gonad_control vs female_gonad_stress | 110361585 | KV878011.1:1658060-1658225 | CE | -0.5462 | 0.988 |
| female_gonad_control vs female_gonad_stress | 102097035 | KV878011.1:1856953-1857032 | CE | -0.47377 | 0.971 |
| female_gonad_control vs female_gonad_stress | 102091752 | KV878011.1:11957459-11957528 | CE | 0.5774 | 1.0 |
| female_hypothalamus_control vs female_hypothalamus_stress | 102083434 | CM007525.1:50528383-50528521 | CE | 0.26024 | 0.958 |
| female_hypothalamus_control vs female_hypothalamus_stress | 102088706 | CM007525.1:20268960-20269198 | CE | -0.5319 | 0.992 |
| female_hypothalamus_control vs female_hypothalamus_stress | 102096569 | CM007525.1:133195257-133195286 | AA | -0.5537 | 0.979 |
| female_hypothalamus_control vs female_hypothalamus_stress | 102085607 | CM007534.1:8527349-8527405 | CE | -0.3728 | 0.951 |
| female_hypothalamus_control vs female_hypothalamus_stress | 102090599 | CM007534.1:18252086-18252100 | AD | -0.46537 | 0.955 |
| female_hypothalamus_control vs female_hypothalamus_stress | 102098594 | CM007535.1:3227169-3227403 | AA | -0.45453 | 0.998 |
| female_hypothalamus_control vs female_hypothalamus_stress | 102098932 | CM007535.1:15101140-15101307 | RI | -0.42642 | 0.977 |
| female_hypothalamus_control vs female_hypothalamus_stress | 102097866 | CM007537.1:16641161-16641174 | RI | -0.63084 | 0.99 |
| female_hypothalamus_control vs female_hypothalamus_stress | 102085568 | CM007537.1:15597310-15597375 | AA | -0.45122 | 0.979 |
| female_hypothalamus_control vs female_hypothalamus_stress | 102084764 | CM007539.1:10562346-10562363 | CE | 0.42504 | 0.98 |
| female_hypothalamus_control vs female_hypothalamus_stress | 102084935 | CM007540.1:8255034-8255293 | RI | 0.49423 | 0.97 |
| female_hypothalamus_control vs female_hypothalamus_stress | 102093197 | CM007541.1:2601490-2601490 | AD | -0.39001 | 0.971 |
| female_hypothalamus_control vs female_hypothalamus_stress | 102093197 | CM007541.1:2601491-2601521 | AD | -0.41074 | 0.981 |
| female_hypothalamus_control vs female_hypothalamus_stress | 102093951 | CM007542.1:2947299-2947321 | AA | -0.62387 | 0.992 |
| female_hypothalamus_control vs female_hypothalamus_stress | 102093485 | CM007542.1:3910477-3910547 | CE | 0.38634 | 0.962 |
| female_hypothalamus_control vs female_hypothalamus_stress | 102085412 | CM007526.1:131182551-131182604 | CE | 0.46049 | 0.989 |
| female_hypothalamus_control vs female_hypothalamus_stress | 102085412 | CM007526.1:131177634-131177687 | CE | 0.44031 | 0.976 |
| female_hypothalamus_control vs female_hypothalamus_stress | 102085412 | CM007526.1:131166341-131166376 | CE | 0.53597 | 0.992 |
| female_hypothalamus_control vs female_hypothalamus_stress | 102085412 | CM007526.1:131164657-131164686 | CE | 0.52653 | 0.989 |
| female_hypothalamus_control vs female_hypothalamus_stress | 102085412 | CM007526.1:131163713-131163766 | CE | 0.54311 | 0.992 |
| female_hypothalamus_control vs female_hypothalamus_stress | 102087735 | CM007526.1:114507878-114508049 | CE | 0.42733 | 0.973 |
| female_hypothalamus_control vs female_hypothalamus_stress | 102090771 | CM007526.1:24607573-24607678 | AD | 0.29535 | 0.953 |
| female_hypothalamus_control vs female_hypothalamus_stress | 103915628 | CM007526.1:5575865-5576266 | CE | 0.40879 | 0.976 |
| female_hypothalamus_control vs female_hypothalamus_stress | 102084352 | CM007526.1:22105709-22105737 | RI | 0.59718 | 0.988 |
| female_hypothalamus_control vs female_hypothalamus_stress | 102088967 | CM007543.1:3392306-3392394 | RI | -0.37816 | 0.975 |
| female_hypothalamus_control vs female_hypothalamus_stress | 102090083 | CM007544.1:765916-766090 | CE | -0.50877 | 0.991 |
| female_hypothalamus_control vs female_hypothalamus_stress | 106146132 | CM007547.1:5268272-5268361 | AA | -0.46396 | 0.978 |
| female_hypothalamus_control vs female_hypothalamus_stress | 102091571 | CM007527.1:31301982-31302232 | CE | 0.41277 | 0.982 |
| female_hypothalamus_control vs female_hypothalamus_stress | 102095597 | CM007527.1:106885152-106885153 | TS | 0.41588 | 0.978 |
| female_hypothalamus_control vs female_hypothalamus_stress | 102092189 | CM007527.1:55078873-55078939 | CE | 0.32435 | 0.964 |
| female_hypothalamus_control vs female_hypothalamus_stress | 102089321 | CM007528.1:60832960-60833028 | AA | 0.3826 | 0.969 |
| female_hypothalamus_control vs female_hypothalamus_stress | 102098752 | CM007528.1:4613-5852 | AD | 0.39012 | 0.964 |
| female_hypothalamus_control vs female_hypothalamus_stress | 102085663 | CM007529.1:9262974-9263061 | CE | -0.39312 | 0.977 |
| female_hypothalamus_control vs female_hypothalamus_stress | 102089918 | CM007529.1:30638128-30638209 | CE | -0.54925 | 0.997 |
| female_hypothalamus_control vs female_hypothalamus_stress | 102087151 | CM007529.1:23927670-23927836 | CE | -0.43834 | 0.976 |
| female_hypothalamus_control vs female_hypothalamus_stress | 102089730 | CM007530.1:1999173-1999280 | CE | 0.47323 | 0.98 |
| female_hypothalamus_control vs female_hypothalamus_stress | 102089203 | CM007530.1:29502009-29502250 | CE | -0.51821 | 0.994 |
| female_hypothalamus_control vs female_hypothalamus_stress | 102084230 | CM007531.1:24032737-24032858 | CE | -0.54645 | 0.99 |
| female_hypothalamus_control vs female_hypothalamus_stress | 102097170 | CM007531.1:5120417-5120620 | CE | -0.41453 | 0.967 |
| female_hypothalamus_control vs female_hypothalamus_stress | 102091346 | CM007531.1:32781562-32782303 | RI | 0.32963 | 0.961 |
| female_hypothalamus_control vs female_hypothalamus_stress | 102095309 | CM007532.1:11798582-11798641 | CE | -0.35209 | 0.971 |
| female_hypothalamus_control vs female_hypothalamus_stress | 102088300 | CM007532.1:16907929-16907995 | AD | 0.53864 | 0.996 |
| female_hypothalamus_control vs female_hypothalamus_stress | 102083979 | CM007533.1:10137747-10137770 | RI | 0.50964 | 0.962 |
| female_hypothalamus_control vs female_hypothalamus_stress | 102085198 | CM007524.1:12243007-12243098 | CE | 0.34973 | 0.963 |
| female_hypothalamus_control vs female_hypothalamus_stress | 104521661 | CM007524.1:5010269-5010291 | CE | -0.40358 | 0.967 |
| female_hypothalamus_control vs female_hypothalamus_stress | 102096323 | KV877987.1:194715-194774 | AA | 0.37432 | 0.969 |
| female_hypothalamus_control vs female_hypothalamus_stress | 110356509 | KV878000.1:224710-224756 | CE | 0.52746 | 0.995 |
| female_hypothalamus_control vs female_hypothalamus_stress | 102094305 | KV878001.1:959028-959191 | CE | 0.38955 | 0.97 |
| female_hypothalamus_control vs female_hypothalamus_stress | 102096431 | KV878011.1:13530545-13530691 | AD | 0.42872 | 0.972 |
| female_hypothalamus_control vs female_hypothalamus_stress | 102093082 | KV878013.1:871935-871985 | AA | -0.48556 | 0.972 |
| female_pituitary_control vs female_pituitary_stress | 102087515 | CM007525.1:1050654-1050680 | CE | 0.44107 | 0.998 |
| female_pituitary_control vs female_pituitary_stress | 102091116 | CM007525.1:59799905-59799908 | AA | 0.62458 | 0.998 |
| female_pituitary_control vs female_pituitary_stress | 105401876 | CM007525.1:72877006-72877086 | CE | 0.60214 | 0.998 |
| female_pituitary_control vs female_pituitary_stress | 102096700 | CM007525.1:133006073-133006175 | CE | 0.42438 | 0.973 |
| female_pituitary_control vs female_pituitary_stress | 102097661 | CM007525.1:52314835-52314994 | CE | -0.36673 | 0.966 |
| female_pituitary_control vs female_pituitary_stress | 102088178 | CM007525.1:186972770-186972777 | AD | -0.55292 | 0.995 |
| female_pituitary_control vs female_pituitary_stress | 102090337 | CM007525.1:199313951-199314356 | AA | 0.35417 | 0.953 |
| female_pituitary_control vs female_pituitary_stress | 102095228 | CM007525.1:48081305-48081319 | AD | 0.45414 | 0.983 |
| female_pituitary_control vs female_pituitary_stress | 102088066 | CM007525.1:55588021-55588110 | CE | 0.36261 | 0.974 |
| female_pituitary_control vs female_pituitary_stress | 102096749 | CM007525.1:98975413-98975442 | AA | 0.42481 | 0.961 |
| female_pituitary_control vs female_pituitary_stress | 102085809 | CM007525.1:134988235-134988291 | CE | 0.3809 | 0.987 |
| female_pituitary_control vs female_pituitary_stress | 102095188 | CM007525.1:69237922-69238053 | CE | 0.35811 | 0.959 |
| female_pituitary_control vs female_pituitary_stress | 102089352 | CM007525.1:69893387-69893415 | AA | 0.32172 | 0.95 |
| female_pituitary_control vs female_pituitary_stress | 102084238 | CM007534.1:4134523-4134629 | AA | 0.40247 | 0.968 |
| female_pituitary_control vs female_pituitary_stress | 102098089 | CM007534.1:900882-901036 | CE | -0.37971 | 0.97 |
| female_pituitary_control vs female_pituitary_stress | 102085836 | CM007535.1:16546131-16546304 | AA | 0.38178 | 0.959 |
| female_pituitary_control vs female_pituitary_stress | 102091392 | CM007536.1:4268940-4269002 | CE | -0.37055 | 0.963 |
| female_pituitary_control vs female_pituitary_stress | 102091392 | CM007536.1:4269081-4269196 | CE | -0.37185 | 0.96 |
| female_pituitary_control vs female_pituitary_stress | 102091392 | CM007536.1:4269720-4269755 | CE | -0.36702 | 0.963 |
| female_pituitary_control vs female_pituitary_stress | 102092413 | CM007537.1:10259343-10259425 | AA | 0.33031 | 0.969 |
| female_pituitary_control vs female_pituitary_stress | 102096541 | CM007537.1:8507445-8508799 | AA | 0.5642 | 0.988 |
| female_pituitary_control vs female_pituitary_stress | 102097332 | CM007537.1:12368435-12368552 | AD | 0.38966 | 0.97 |
| female_pituitary_control vs female_pituitary_stress | 102095946 | CM007537.1:12539802-12539911 | AA | -0.41462 | 1.0 |
| female_pituitary_control vs female_pituitary_stress | 106145708 | CM007537.1:15867491-15867970 | AD | 0.34646 | 0.978 |
| female_pituitary_control vs female_pituitary_stress | 102086043 | CM007538.1:2856682-2856835 | CE | -0.34164 | 0.967 |
| female_pituitary_control vs female_pituitary_stress | 102094726 | CM007538.1:1050391-1050506 | CE | 0.3478 | 0.957 |
| female_pituitary_control vs female_pituitary_stress | 103912455 | CM007539.1:1188798-1188837 | AD | -0.31944 | 0.954 |
| female_pituitary_control vs female_pituitary_stress | 102093545 | CM007539.1:8496546-8496597 | AD | -0.27564 | 0.961 |
| female_pituitary_control vs female_pituitary_stress | 102088368 | CM007539.1:9860739-9860890 | CE | -0.36903 | 0.951 |
| female_pituitary_control vs female_pituitary_stress | 104022714 | CM007540.1:619765-619827 | CE | 0.22324 | 1.0 |
| female_pituitary_control vs female_pituitary_stress | 102088530 | CM007540.1:4869020-4869196 | CE | -0.48839 | 0.997 |
| female_pituitary_control vs female_pituitary_stress | 102085103 | CM007541.1:9073788-9074156 | RI | 0.41948 | 0.974 |
| female_pituitary_control vs female_pituitary_stress | 102095052 | CM007542.1:2403387-2403608 | CE | 0.26586 | 0.969 |
| female_pituitary_control vs female_pituitary_stress | 102090571 | CM007542.1:7630028-7630028 | AD | 0.4429 | 0.979 |
| female_pituitary_control vs female_pituitary_stress | 102090571 | CM007542.1:7630029-7630087 | AD | 0.45016 | 0.989 |
| female_pituitary_control vs female_pituitary_stress | 102092349 | CM007542.1:670607-670648 | CE | 0.55203 | 0.993 |
| female_pituitary_control vs female_pituitary_stress | 102084088 | CM007542.1:3314281-3314374 | CE | -0.16638 | 0.969 |
| female_pituitary_control vs female_pituitary_stress | 102096825 | CM007526.1:45369874-45369948 | CE | -0.42159 | 0.974 |
| female_pituitary_control vs female_pituitary_stress | 102088550 | CM007526.1:16363004-16363045 | CE | 0.35814 | 0.963 |
| female_pituitary_control vs female_pituitary_stress | 102092361 | CM007526.1:122839471-122839561 | CE | 0.27813 | 0.963 |
| female_pituitary_control vs female_pituitary_stress | 102096498 | CM007526.1:72828923-72829087 | CE | -0.1178 | 0.957 |
| female_pituitary_control vs female_pituitary_stress | 102090239 | CM007526.1:20600454-20600785 | CE | 0.42781 | 0.999 |
| female_pituitary_control vs female_pituitary_stress | 102084143 | CM007526.1:86039989-86040048 | AA | -0.39512 | 0.972 |
| female_pituitary_control vs female_pituitary_stress | 102094379 | CM007526.1:31091261-31091278 | AD | -0.49396 | 0.987 |
| female_pituitary_control vs female_pituitary_stress | 102083948 | CM007526.1:34611547-34611650 | CE | -0.34696 | 0.972 |
| female_pituitary_control vs female_pituitary_stress | 102084570 | CM007526.1:72552226-72552353 | AA | 0.39376 | 0.967 |
| female_pituitary_control vs female_pituitary_stress | 102084570 | CM007526.1:72552514-72552514 | AD | 0.49747 | 0.991 |
| female_pituitary_control vs female_pituitary_stress | 102084369 | CM007526.1:3009066-3009091 | CE | 0.67106 | 1.0 |
| female_pituitary_control vs female_pituitary_stress | 102092628 | CM007543.1:4697777-4697789 | AD | -0.54514 | 0.999 |
| female_pituitary_control vs female_pituitary_stress | 102094555 | CM007543.1:897243-897296 | CE | 0.75793 | 0.999 |
| female_pituitary_control vs female_pituitary_stress | 102091635 | CM007544.1:5499598-5499701 | CE | 0.64044 | 0.998 |
| female_pituitary_control vs female_pituitary_stress | 102091635 | CM007544.1:5493349-5493584 | AA | -0.57515 | 0.995 |
| female_pituitary_control vs female_pituitary_stress | 102091635 | CM007544.1:5491040-5491220 | CE | 0.22996 | 0.977 |
| female_pituitary_control vs female_pituitary_stress | 102091635 | CM007544.1:5490894-5490966 | CE | 0.21041 | 0.967 |
| female_pituitary_control vs female_pituitary_stress | 102098083 | CM007544.1:1531290-1531417 | CE | -0.21503 | 0.954 |
| female_pituitary_control vs female_pituitary_stress | 102088149 | CM007546.1:2919903-2920073 | CE | -0.36856 | 0.979 |
| female_pituitary_control vs female_pituitary_stress | 102098180 | CM007547.1:2986767-2986808 | CE | 0.46115 | 0.987 |
| female_pituitary_control vs female_pituitary_stress | 102094060 | CM007547.1:239329-239431 | CE | 0.40747 | 0.964 |
| female_pituitary_control vs female_pituitary_stress | 102086868 | CM007547.1:2528742-2529038 | AA | 0.37469 | 0.964 |
| female_pituitary_control vs female_pituitary_stress | 102086868 | CM007547.1:2532500-2532640 | CE | -0.53079 | 0.957 |
| female_pituitary_control vs female_pituitary_stress | 102084175 | CM007550.1:966115-966239 | CE | 0.3892 | 0.969 |
| female_pituitary_control vs female_pituitary_stress | 102097848 | CM007550.1:3515759-3515773 | AD | -0.42603 | 0.964 |
| female_pituitary_control vs female_pituitary_stress | 102089128 | CM007550.1:2415175-2415854 | AD | 0.33748 | 0.951 |
| female_pituitary_control vs female_pituitary_stress | 102088697 | CM007527.1:44153164-44153366 | CE | -0.27671 | 0.993 |
| female_pituitary_control vs female_pituitary_stress | 102084408 | CM007527.1:80098138-80098404 | CE | 0.15021 | 0.96 |
| female_pituitary_control vs female_pituitary_stress | 102095706 | CM007527.1:46869090-46869230 | CE | -0.10526 | 0.967 |
| female_pituitary_control vs female_pituitary_stress | 102093700 | CM007527.1:106273696-106273889 | RI | -0.35438 | 1.0 |
| female_pituitary_control vs female_pituitary_stress | 110355187 | CM007527.1:507844-507872 | AD | -0.56605 | 0.995 |
| female_pituitary_control vs female_pituitary_stress | 102096691 | CM007527.1:65171832-65171858 | AD | -0.41242 | 0.951 |
| female_pituitary_control vs female_pituitary_stress | 102094099 | CM007528.1:27890532-27890540 | AD | 0.30636 | 0.961 |
| female_pituitary_control vs female_pituitary_stress | 102095735 | CM007528.1:15990576-15990632 | CE | 0.33121 | 0.975 |
| female_pituitary_control vs female_pituitary_stress | 102095733 | CM007528.1:13642533-13642601 | AA | -0.4019 | 0.978 |
| female_pituitary_control vs female_pituitary_stress | 110356341 | CM007528.1:12023521-12023601 | CE | 0.24885 | 0.961 |
| female_pituitary_control vs female_pituitary_stress | 110356341 | CM007528.1:12037501-12037587 | CE | -0.36232 | 0.966 |
| female_pituitary_control vs female_pituitary_stress | 102087100 | CM007528.1:58298436-58298663 | CE | 0.12782 | 0.978 |
| female_pituitary_control vs female_pituitary_stress | 102098275 | CM007528.1:52695294-52695297 | AA | -0.3347 | 0.953 |
| female_pituitary_control vs female_pituitary_stress | 102083833 | CM007529.1:10044758-10044874 | CE | 0.42189 | 0.965 |
| female_pituitary_control vs female_pituitary_stress | 102098756 | CM007529.1:18740840-18741615 | AD | 0.4883 | 0.98 |
| female_pituitary_control vs female_pituitary_stress | 102083807 | CM007529.1:46982665-46982751 | AA | 0.49529 | 0.966 |
| female_pituitary_control vs female_pituitary_stress | 102096550 | CM007530.1:26224643-26224702 | CE | 0.36382 | 0.97 |
| female_pituitary_control vs female_pituitary_stress | 102091060 | CM007530.1:15569048-15569068 | CE | 0.48443 | 0.971 |
| female_pituitary_control vs female_pituitary_stress | 102091355 | CM007530.1:18396516-18396548 | CE | -0.45629 | 0.988 |
| female_pituitary_control vs female_pituitary_stress | 102089159 | CM007530.1:34813327-34813437 | CE | -0.36895 | 0.964 |
| female_pituitary_control vs female_pituitary_stress | 102097566 | CM007531.1:8007731-8008415 | AD | 0.52308 | 0.981 |
| female_pituitary_control vs female_pituitary_stress | 102093135 | CM007531.1:19016788-19016835 | AA | 0.40363 | 0.952 |
| female_pituitary_control vs female_pituitary_stress | 102085385 | CM007531.1:14765898-14765946 | CE | -0.58427 | 0.994 |
| female_pituitary_control vs female_pituitary_stress | 102083922 | CM007531.1:24074903-24075800 | AA | 0.74727 | 1.0 |
| female_pituitary_control vs female_pituitary_stress | 102091432 | CM007531.1:23186188-23186239 | CE | 0.34502 | 0.965 |
| female_pituitary_control vs female_pituitary_stress | 102091432 | CM007531.1:23186780-23186853 | CE | 0.34447 | 0.969 |
| female_pituitary_control vs female_pituitary_stress | 102087352 | CM007531.1:19153944-19153950 | TE | -0.41093 | 0.974 |
| female_pituitary_control vs female_pituitary_stress | 102087352 | CM007531.1:19153951-19153953 | TE | 0.41515 | 0.957 |
| female_pituitary_control vs female_pituitary_stress | 102097377 | CM007532.1:13293089-13293274 | CE | -0.42441 | 0.984 |
| female_pituitary_control vs female_pituitary_stress | 102098898 | CM007533.1:10673387-10673454 | CE | -0.42617 | 0.971 |
| female_pituitary_control vs female_pituitary_stress | 102086708 | CM007533.1:19843036-19843123 | CE | 0.43112 | 0.987 |
| female_pituitary_control vs female_pituitary_stress | 102087048 | CM007524.1:1120761-1120901 | CE | 0.40955 | 0.971 |
| female_pituitary_control vs female_pituitary_stress | 102090591 | CM007524.1:24727852-24727901 | CE | -0.48996 | 0.998 |
| female_pituitary_control vs female_pituitary_stress | 102088929 | CM007524.1:5934106-5934356 | CE | 0.39944 | 0.96 |
| female_pituitary_control vs female_pituitary_stress | 102093178 | CM007524.1:32515124-32515150 | CE | 0.43388 | 0.975 |
| female_pituitary_control vs female_pituitary_stress | 102090392 | KV877993.1:1287408-1287533 | CE | -0.34238 | 0.957 |
| female_pituitary_control vs female_pituitary_stress | 102090392 | KV877993.1:1287151-1287276 | CE | -0.33304 | 0.956 |
| female_pituitary_control vs female_pituitary_stress | 105411610 | KV877994.1:1637599-1637668 | CE | -0.59044 | 0.996 |
| female_pituitary_control vs female_pituitary_stress | 102095595 | KV877999.1:2526570-2526590 | CE | 0.41449 | 0.975 |
| female_pituitary_control vs female_pituitary_stress | 102088182 | KV878000.1:6614501-6614764 | CE | -0.40461 | 0.954 |
| female_pituitary_control vs female_pituitary_stress | 102091925 | KV878001.1:1026659-1026705 | AD | 0.41534 | 0.98 |
| female_pituitary_control vs female_pituitary_stress | 102093886 | KV878003.1:844474-844582 | RI | 0.58001 | 1.0 |
| female_pituitary_control vs female_pituitary_stress | 102093886 | KV878003.1:845934-846170 | RI | -0.37311 | 0.98 |
| female_pituitary_control vs female_pituitary_stress | 105408022 | KV878013.1:1003877-1003957 | CE | -0.36012 | 0.991 |
| male_gonad_control vs male_gonad_stress | 102098904 | CM007525.1:50565625-50565757 | CE | 0.36641 | 0.959 |
| male_gonad_control vs male_gonad_stress | 102086997 | CM007525.1:177653687-177653712 | CE | 0.29269 | 0.972 |
| male_gonad_control vs male_gonad_stress | 110358081 | CM007525.1:186730316-186730562 | CE | 0.39016 | 0.967 |
| male_gonad_control vs male_gonad_stress | 102097369 | CM007525.1:52809243-52809307 | CE | 0.52149 | 0.981 |
| male_gonad_control vs male_gonad_stress | 102086283 | CM007534.1:1828796-1828823 | CE | 0.34654 | 0.953 |
| male_gonad_control vs male_gonad_stress | 102096684 | CM007536.1:6904412-6904498 | RI | -0.37175 | 0.95 |
| male_gonad_control vs male_gonad_stress | 102096732 | CM007537.1:8488157-8488234 | CE | -0.36667 | 0.956 |
| male_gonad_control vs male_gonad_stress | 102095854 | CM007537.1:19133581-19133660 | AA | 0.5643 | 0.977 |
| male_gonad_control vs male_gonad_stress | 102088507 | CM007538.1:2716734-2716767 | AA | 0.30373 | 0.95 |
| male_gonad_control vs male_gonad_stress | 102093249 | CM007539.1:12101873-12101938 | CE | 0.35294 | 0.966 |
| male_gonad_control vs male_gonad_stress | 110355067 | CM007540.1:10281323-10282471 | CE | -0.358 | 0.954 |
| male_gonad_control vs male_gonad_stress | 102091335 | CM007540.1:6170339-6170361 | RI | 0.54231 | 0.981 |
| male_gonad_control vs male_gonad_stress | 106146053 | CM007540.1:2224444-2224497 | CE | 0.41964 | 0.979 |
| male_gonad_control vs male_gonad_stress | 106146053 | CM007540.1:2225176-2225229 | CE | 0.4039 | 0.972 |
| male_gonad_control vs male_gonad_stress | 102086211 | CM007542.1:5313689-5313741 | CE | 0.32556 | 0.951 |
| male_gonad_control vs male_gonad_stress | 102092763 | CM007542.1:4159834-4159861 | TS | -0.51597 | 0.992 |
| male_gonad_control vs male_gonad_stress | 102092763 | CM007542.1:4156340-4156598 | TS | 0.50795 | 0.989 |
| male_gonad_control vs male_gonad_stress | 102092590 | CM007542.1:4184865-4184897 | AD | -0.51606 | 0.99 |
| male_gonad_control vs male_gonad_stress | 102093951 | CM007542.1:2947299-2947321 | AA | -0.58726 | 0.979 |
| male_gonad_control vs male_gonad_stress | 102095142 | CM007526.1:21931503-21931659 | CE | -0.45404 | 0.98 |
| male_gonad_control vs male_gonad_stress | 102084031 | CM007526.1:93219666-93219929 | RI | -0.42082 | 0.974 |
| male_gonad_control vs male_gonad_stress | 102094063 | CM007546.1:4094396-4094396 | AA | 0.44937 | 0.97 |
| male_gonad_control vs male_gonad_stress | 102085052 | CM007546.1:2438402-2438601 | AD | -0.37949 | 0.951 |
| male_gonad_control vs male_gonad_stress | 104914348 | CM007547.1:1379643-1379788 | CE | 0.44934 | 0.985 |
| male_gonad_control vs male_gonad_stress | 102090846 | CM007527.1:87926808-87926852 | AA | -0.57827 | 0.993 |
| male_gonad_control vs male_gonad_stress | 102090542 | CM007527.1:46655425-46655607 | CE | 0.48506 | 0.975 |
| male_gonad_control vs male_gonad_stress | 102092374 | CM007527.1:55012175-55012247 | CE | 0.31359 | 0.963 |
| male_gonad_control vs male_gonad_stress | 102092436 | CM007527.1:850531-850648 | RI | -0.75317 | 0.998 |
| male_gonad_control vs male_gonad_stress | 104034471 | CM007527.1:9770080-9770209 | CE | 0.4331 | 0.968 |
| male_gonad_control vs male_gonad_stress | 102084328 | CM007528.1:200251-200413 | AD | -0.65532 | 0.998 |
| male_gonad_control vs male_gonad_stress | 110365763 | CM007523.1:3072242-3072308 | CE | -0.419 | 0.955 |
| male_gonad_control vs male_gonad_stress | 102097852 | CM007523.1:364672-365956 | AD | 0.572 | 0.989 |
| male_gonad_control vs male_gonad_stress | 110360169 | CM007530.1:20926377-20926538 | CE | -0.28929 | 0.954 |
| male_gonad_control vs male_gonad_stress | 102087010 | CM007530.1:235288-235360 | RI | -0.35769 | 0.965 |
| male_gonad_control vs male_gonad_stress | 102040246 | CM007531.1:18592895-18592972 | CE | -0.52917 | 0.959 |
| male_gonad_control vs male_gonad_stress | 102083695 | CM007531.1:5610302-5612218 | RI | 0.38636 | 0.95 |
| male_gonad_control vs male_gonad_stress | 102097377 | CM007532.1:13292953-13293068 | RI | 0.4598 | 0.977 |
| male_gonad_control vs male_gonad_stress | 102097377 | CM007532.1:13293069-13293088 | AA | 0.43728 | 0.971 |
| male_gonad_control vs male_gonad_stress | 102084074 | CM007532.1:14786453-14787203 | TE | -0.50291 | 0.992 |
| male_gonad_control vs male_gonad_stress | 102084074 | CM007532.1:14786451-14786452 | TE | 0.48829 | 0.992 |
| male_gonad_control vs male_gonad_stress | 102089105 | CM007533.1:20168947-20169011 | CE | 0.26116 | 0.975 |
| male_gonad_control vs male_gonad_stress | 102098881 | CM007533.1:20731988-20732192 | CE | -0.42628 | 0.982 |
| male_gonad_control vs male_gonad_stress | 102091285 | CM007533.1:7557249-7557629 | CE | -0.43525 | 0.978 |
| male_gonad_control vs male_gonad_stress | 102093887 | KV878002.1:1017201-1017323 | AA | 0.46744 | 0.963 |
| male_gonad_control vs male_gonad_stress | 110364063 | KV878017.1:3236262-3236351 | CE | 0.52611 | 0.996 |
| male_hypothalamus_control vs male_hypothalamus_stress | 102088390 | CM007525.1:195274189-195274457 | RI | 0.33855 | 0.959 |
| male_hypothalamus_control vs male_hypothalamus_stress | 102086227 | CM007525.1:78705348-78705474 | CE | 0.56837 | 0.999 |
| male_hypothalamus_control vs male_hypothalamus_stress | 102090337 | CM007525.1:199316330-199316345 | CE | 0.73814 | 0.999 |
| male_hypothalamus_control vs male_hypothalamus_stress | 102092451 | CM007525.1:49375017-49375093 | CE | 0.38096 | 0.973 |
| male_hypothalamus_control vs male_hypothalamus_stress | 106145849 | CM007525.1:48648127-48648683 | RI | 0.49648 | 0.992 |
| male_hypothalamus_control vs male_hypothalamus_stress | 102091457 | CM007525.1:129454716-129455025 | RI | -0.45055 | 0.976 |
| male_hypothalamus_control vs male_hypothalamus_stress | 102096928 | CM007525.1:73848992-73849026 | AD | -0.62901 | 0.994 |
| male_hypothalamus_control vs male_hypothalamus_stress | 106145910 | CM007525.1:117073402-117073489 | CE | -0.38447 | 0.967 |
| male_hypothalamus_control vs male_hypothalamus_stress | 102095370 | CM007534.1:362101-362683 | CE | 0.47419 | 0.987 |
| male_hypothalamus_control vs male_hypothalamus_stress | 102089699 | CM007534.1:7691693-7691776 | CE | -0.72157 | 0.998 |
| male_hypothalamus_control vs male_hypothalamus_stress | 102090599 | CM007534.1:18243058-18243113 | AA | 0.45518 | 0.995 |
| male_hypothalamus_control vs male_hypothalamus_stress | 102098594 | CM007535.1:3240799-3241158 | AA | -0.37965 | 0.96 |
| male_hypothalamus_control vs male_hypothalamus_stress | 102085302 | CM007535.1:16333531-16333612 | AA | 0.47583 | 0.998 |
| male_hypothalamus_control vs male_hypothalamus_stress | 102085568 | CM007537.1:15598745-15599675 | RI | 0.48022 | 0.985 |
| male_hypothalamus_control vs male_hypothalamus_stress | 102089642 | CM007537.1:17220600-17220683 | CE | 0.66121 | 0.999 |
| male_hypothalamus_control vs male_hypothalamus_stress | 106145708 | CM007537.1:15867491-15867970 | AD | 0.58922 | 0.998 |
| male_hypothalamus_control vs male_hypothalamus_stress | 102084977 | CM007538.1:4222316-4222655 | TE | -0.53083 | 0.999 |
| male_hypothalamus_control vs male_hypothalamus_stress | 102084977 | CM007538.1:4222656-4222658 | TE | 0.54176 | 1.0 |
| male_hypothalamus_control vs male_hypothalamus_stress | 102090947 | CM007539.1:8686221-8686415 | RI | -0.31674 | 0.976 |
| male_hypothalamus_control vs male_hypothalamus_stress | 102090698 | CM007539.1:5096624-5096719 | CE | 0.4321 | 0.975 |
| male_hypothalamus_control vs male_hypothalamus_stress | 102090698 | CM007539.1:5097097-5097268 | CE | 0.44734 | 0.979 |
| male_hypothalamus_control vs male_hypothalamus_stress | 102090698 | CM007539.1:5097763-5097894 | CE | 0.57215 | 0.998 |
| male_hypothalamus_control vs male_hypothalamus_stress | 102085923 | CM007539.1:12923659-12923715 | AD | 0.49894 | 0.99 |
| male_hypothalamus_control vs male_hypothalamus_stress | 102091885 | CM007540.1:8597041-8597103 | CE | 0.43037 | 0.959 |
| male_hypothalamus_control vs male_hypothalamus_stress | 102089640 | CM007541.1:8710829-8711700 | RI | 0.31211 | 0.954 |
| male_hypothalamus_control vs male_hypothalamus_stress | 102094350 | CM007542.1:6987363-6987397 | AA | 0.42948 | 0.956 |
| male_hypothalamus_control vs male_hypothalamus_stress | 102086287 | CM007542.1:7219486-7219544 | AA | 0.50416 | 0.99 |
| male_hypothalamus_control vs male_hypothalamus_stress | 102092335 | CM007526.1:113549045-113549066 | AA | -0.66613 | 1.0 |
| male_hypothalamus_control vs male_hypothalamus_stress | 102084933 | CM007526.1:6319844-6319883 | CE | 0.53497 | 0.991 |
| male_hypothalamus_control vs male_hypothalamus_stress | 102090174 | CM007526.1:41275867-41275885 | AA | 0.28424 | 0.973 |
| male_hypothalamus_control vs male_hypothalamus_stress | 102084143 | CM007526.1:86039128-86039170 | AD | -0.39095 | 0.976 |
| male_hypothalamus_control vs male_hypothalamus_stress | 102084570 | CM007526.1:72550030-72552225 | RI | 0.35015 | 0.968 |
| male_hypothalamus_control vs male_hypothalamus_stress | 102084570 | CM007526.1:72552514-72552514 | AD | 0.4631 | 0.979 |
| male_hypothalamus_control vs male_hypothalamus_stress | 102084570 | CM007526.1:72552515-72552630 | RI | 0.48243 | 0.989 |
| male_hypothalamus_control vs male_hypothalamus_stress | 102091635 | CM007544.1:5495979-5496058 | CE | 0.72869 | 1.0 |
| male_hypothalamus_control vs male_hypothalamus_stress | 102087006 | CM007544.1:310883-310896 | AD | -0.74244 | 1.0 |
| male_hypothalamus_control vs male_hypothalamus_stress | 102093138 | CM007544.1:4715863-4715898 | AA | -0.37341 | 0.956 |
| male_hypothalamus_control vs male_hypothalamus_stress | 102087608 | CM007544.1:1557915-1559913 | AA | 0.61091 | 0.973 |
| male_hypothalamus_control vs male_hypothalamus_stress | 102087608 | CM007544.1:1559914-1560246 | CE | 0.51414 | 0.971 |
| male_hypothalamus_control vs male_hypothalamus_stress | 102087608 | CM007544.1:1560247-1565077 | RI | 0.49712 | 0.961 |
| male_hypothalamus_control vs male_hypothalamus_stress | 102087608 | CM007544.1:1565078-1565081 | AA | 0.45409 | 0.951 |
| male_hypothalamus_control vs male_hypothalamus_stress | 102096692 | CM007546.1:5439174-5439231 | CE | 0.28717 | 0.951 |
| male_hypothalamus_control vs male_hypothalamus_stress | 102097419 | CM007546.1:5605760-5607193 | TE | -0.53391 | 0.995 |
| male_hypothalamus_control vs male_hypothalamus_stress | 102097419 | CM007546.1:5607194-5607196 | TE | 0.53328 | 0.998 |
| male_hypothalamus_control vs male_hypothalamus_stress | 102098180 | CM007547.1:2986767-2986808 | CE | 0.43546 | 0.989 |
| male_hypothalamus_control vs male_hypothalamus_stress | 102086160 | CM007551.1:864538-864942 | RI | -0.43182 | 0.972 |
| male_hypothalamus_control vs male_hypothalamus_stress | 102086160 | CM007551.1:864448-864537 | CE | -0.44769 | 0.978 |
| male_hypothalamus_control vs male_hypothalamus_stress | 102088688 | CM007527.1:24808847-24808907 | CE | -0.42773 | 0.977 |
| male_hypothalamus_control vs male_hypothalamus_stress | 102091617 | CM007527.1:26758381-26758399 | AD | 0.5107 | 0.956 |
| male_hypothalamus_control vs male_hypothalamus_stress | 102093328 | CM007527.1:111265894-111266028 | CE | -0.32129 | 0.96 |
| male_hypothalamus_control vs male_hypothalamus_stress | 102092123 | CM007527.1:30800224-30800290 | CE | -0.49442 | 0.99 |
| male_hypothalamus_control vs male_hypothalamus_stress | 102089252 | CM007527.1:24548935-24548935 | AD | 0.54987 | 0.996 |
| male_hypothalamus_control vs male_hypothalamus_stress | 102088088 | CM007527.1:1036146-1036448 | RI | -0.47951 | 0.983 |
| male_hypothalamus_control vs male_hypothalamus_stress | 102084328 | CM007528.1:200251-200413 | AD | -0.78261 | 1.0 |
| male_hypothalamus_control vs male_hypothalamus_stress | 102085483 | CM007528.1:24724892-24724938 | AA | 0.4234 | 0.972 |
| male_hypothalamus_control vs male_hypothalamus_stress | 102095379 | CM007523.1:11971917-11971970 | CE | -0.4451 | 0.974 |
| male_hypothalamus_control vs male_hypothalamus_stress | 102087749 | CM007529.1:24140245-24140247 | AA | 0.35082 | 0.953 |
| male_hypothalamus_control vs male_hypothalamus_stress | 102090171 | CM007530.1:10561460-10561863 | RI | -0.49137 | 0.992 |
| male_hypothalamus_control vs male_hypothalamus_stress | 102095997 | CM007530.1:4955797-4956018 | TE | 0.48496 | 0.989 |
| male_hypothalamus_control vs male_hypothalamus_stress | 102095997 | CM007530.1:4956019-4956072 | TE | -0.47922 | 0.986 |
| male_hypothalamus_control vs male_hypothalamus_stress | 102093658 | CM007530.1:2534040-2534057 | CE | 0.7244 | 0.999 |
| male_hypothalamus_control vs male_hypothalamus_stress | 102093658 | CM007530.1:2537921-2537968 | CE | 0.63697 | 0.998 |
| male_hypothalamus_control vs male_hypothalamus_stress | 102093658 | CM007530.1:2544887-2544934 | CE | 0.42965 | 0.973 |
| male_hypothalamus_control vs male_hypothalamus_stress | 102088201 | CM007531.1:17714849-17715236 | RI | 0.23165 | 0.972 |
| male_hypothalamus_control vs male_hypothalamus_stress | 102089841 | CM007531.1:243741-243839 | CE | 0.43342 | 0.966 |
| male_hypothalamus_control vs male_hypothalamus_stress | 102097571 | CM007532.1:23222613-23222632 | RI | 0.35228 | 0.974 |
| male_hypothalamus_control vs male_hypothalamus_stress | 102097377 | CM007532.1:13292767-13292952 | CE | -0.3554 | 0.96 |
| male_hypothalamus_control vs male_hypothalamus_stress | 102084643 | CM007532.1:22281571-22281820 | AF | -0.48987 | 0.977 |
| male_hypothalamus_control vs male_hypothalamus_stress | 102088300 | CM007532.1:16918015-16918099 | CE | -0.29925 | 0.955 |
| male_hypothalamus_control vs male_hypothalamus_stress | 102094943 | CM007532.1:10555223-10555247 | AD | -0.44881 | 0.954 |
| male_hypothalamus_control vs male_hypothalamus_stress | 102087276 | CM007533.1:14218585-14218649 | CE | -0.40457 | 0.954 |
| male_hypothalamus_control vs male_hypothalamus_stress | 102091278 | CM007533.1:13086616-13086616 | TS | -0.40273 | 0.988 |
| male_hypothalamus_control vs male_hypothalamus_stress | 102091278 | CM007533.1:13086618-13086858 | TS | 0.6488 | 1.0 |
| male_hypothalamus_control vs male_hypothalamus_stress | 102098416 | CM007533.1:14145644-14145675 | CE | -0.43896 | 0.97 |
| male_hypothalamus_control vs male_hypothalamus_stress | 110362363 | CM007533.1:2063884-2063906 | AA | 0.368 | 0.984 |
| male_hypothalamus_control vs male_hypothalamus_stress | 102092245 | CM007533.1:12589607-12589684 | CE | 0.24445 | 0.961 |
| male_hypothalamus_control vs male_hypothalamus_stress | 102085148 | CM007524.1:2453448-2454549 | AD | -0.42097 | 0.977 |
| male_hypothalamus_control vs male_hypothalamus_stress | 102093302 | CM007524.1:19620105-19621355 | RI | 0.39427 | 0.99 |
| male_hypothalamus_control vs male_hypothalamus_stress | 102090375 | CM007524.1:15177231-15177317 | RI | -0.43246 | 0.966 |
| male_hypothalamus_control vs male_hypothalamus_stress | 102086113 | CM007524.1:27610462-27610581 | AA | -0.30013 | 0.956 |
| male_hypothalamus_control vs male_hypothalamus_stress | 102091397 | CM007524.1:21070644-21070719 | CE | 0.31048 | 0.95 |
| male_hypothalamus_control vs male_hypothalamus_stress | 102086537 | KV877990.1:1467043-1467048 | CE | 0.59954 | 0.996 |
| male_hypothalamus_control vs male_hypothalamus_stress | 102088881 | KV877991.1:1151453-1151488 | CE | 0.44818 | 0.987 |
| male_hypothalamus_control vs male_hypothalamus_stress | 103895355 | KV877998.1:1940450-1940487 | AA | 0.48467 | 0.996 |
| male_hypothalamus_control vs male_hypothalamus_stress | 102095769 | KV877999.1:3282866-3282906 | CE | 0.46781 | 0.989 |
| male_hypothalamus_control vs male_hypothalamus_stress | 102091929 | KV877999.1:5196722-5196794 | AA | -0.35742 | 0.969 |
| male_hypothalamus_control vs male_hypothalamus_stress | 102097653 | KV877999.1:520737-522313 | RI | 0.29585 | 0.962 |
| male_hypothalamus_control vs male_hypothalamus_stress | 102085700 | KV878003.1:6460487-6460519 | CE | 0.59353 | 0.97 |
| male_hypothalamus_control vs male_hypothalamus_stress | 102094490 | KV878004.1:3082306-3082345 | AD | 0.45908 | 0.972 |
| male_pituitary_control vs male_pituitary_stress | 102091119 | CM007525.1:174970644-174970717 | CE | -0.26345 | 0.952 |
| male_pituitary_control vs male_pituitary_stress | 102091538 | CM007525.1:67148785-67148975 | CE | 0.48904 | 0.99 |
| male_pituitary_control vs male_pituitary_stress | 102088097 | CM007525.1:40351219-40351246 | AA | 0.42837 | 0.977 |
| male_pituitary_control vs male_pituitary_stress | 102094712 | CM007525.1:176925214-176925261 | CE | -0.37145 | 0.951 |
| male_pituitary_control vs male_pituitary_stress | 102090035 | CM007525.1:81988467-81988520 | CE | -0.42966 | 0.969 |
| male_pituitary_control vs male_pituitary_stress | 102090756 | CM007525.1:59725118-59725284 | CE | 0.13115 | 0.968 |
| male_pituitary_control vs male_pituitary_stress | 102090756 | CM007525.1:59730544-59730700 | CE | 0.1425 | 0.998 |
| male_pituitary_control vs male_pituitary_stress | 102090756 | CM007525.1:59732057-59732245 | CE | 0.11605 | 0.984 |
| male_pituitary_control vs male_pituitary_stress | 102090756 | CM007525.1:59733672-59733806 | CE | 0.12208 | 0.989 |
| male_pituitary_control vs male_pituitary_stress | 102090756 | CM007525.1:59738869-59739036 | CE | 0.12246 | 0.973 |
| male_pituitary_control vs male_pituitary_stress | 102090756 | CM007525.1:59742074-59742213 | CE | 0.12378 | 0.979 |
| male_pituitary_control vs male_pituitary_stress | 102090756 | CM007525.1:59745762-59745898 | CE | 0.11004 | 0.973 |
| male_pituitary_control vs male_pituitary_stress | 102096569 | CM007525.1:133193502-133193621 | AA | 0.49681 | 0.991 |
| male_pituitary_control vs male_pituitary_stress | 102087178 | CM007525.1:71209438-71209521 | CE | 0.5087 | 0.995 |
| male_pituitary_control vs male_pituitary_stress | 102097480 | CM007525.1:98350496-98350644 | CE | -0.45919 | 0.984 |
| male_pituitary_control vs male_pituitary_stress | 102090452 | CM007525.1:48648978-48649147 | CE | -0.44686 | 0.97 |
| male_pituitary_control vs male_pituitary_stress | 102094617 | CM007525.1:129636742-129636820 | CE | 0.37565 | 0.981 |
| male_pituitary_control vs male_pituitary_stress | 102084196 | CM007525.1:35974768-35974779 | AD | 0.33613 | 0.964 |
| male_pituitary_control vs male_pituitary_stress | 102098015 | CM007534.1:4097813-4097946 | CE | 0.57183 | 0.998 |
| male_pituitary_control vs male_pituitary_stress | 102084780 | CM007535.1:4674605-4674624 | RI | 0.48939 | 0.992 |
| male_pituitary_control vs male_pituitary_stress | 102097851 | CM007535.1:16386652-16386753 | CE | 0.27969 | 0.98 |
| male_pituitary_control vs male_pituitary_stress | 102097851 | CM007535.1:16374024-16374110 | CE | -0.40326 | 0.972 |
| male_pituitary_control vs male_pituitary_stress | 102095514 | CM007535.1:5724410-5724410 | AD | -0.76342 | 0.999 |
| male_pituitary_control vs male_pituitary_stress | 102098891 | CM007535.1:10803759-10803914 | RI | 0.56937 | 0.999 |
| male_pituitary_control vs male_pituitary_stress | 102091392 | CM007536.1:4273411-4273446 | CE | 0.29514 | 0.96 |
| male_pituitary_control vs male_pituitary_stress | 102085568 | CM007537.1:15596933-15597309 | RI | 0.4251 | 0.979 |
| male_pituitary_control vs male_pituitary_stress | 102093071 | CM007537.1:1648587-1648691 | RI | 0.32291 | 0.974 |
| male_pituitary_control vs male_pituitary_stress | 106145708 | CM007537.1:15868296-15868338 | AA | -0.35952 | 0.957 |
| male_pituitary_control vs male_pituitary_stress | 102088507 | CM007538.1:2693177-2694450 | CE | -0.5409 | 0.981 |
| male_pituitary_control vs male_pituitary_stress | 102083892 | CM007539.1:3366607-3366873 | RI | -0.41768 | 0.976 |
| male_pituitary_control vs male_pituitary_stress | 102098326 | CM007539.1:71281-71562 | RI | 0.47456 | 0.978 |
| male_pituitary_control vs male_pituitary_stress | 102084176 | CM007541.1:8727750-8727753 | AD | 0.79119 | 1.0 |
| male_pituitary_control vs male_pituitary_stress | 102084176 | CM007541.1:8727694-8727749 | AD | 0.72637 | 0.998 |
| male_pituitary_control vs male_pituitary_stress | 103896199 | CM007526.1:2763199-2763465 | CE | -0.31133 | 0.961 |
| male_pituitary_control vs male_pituitary_stress | 102086718 | CM007526.1:15455242-15456126 | CE | 0.45491 | 0.989 |
| male_pituitary_control vs male_pituitary_stress | 102086718 | CM007526.1:15454739-15454909 | CE | 0.52543 | 0.999 |
| male_pituitary_control vs male_pituitary_stress | 102084933 | CM007526.1:6315033-6315118 | CE | -0.74848 | 1.0 |
| male_pituitary_control vs male_pituitary_stress | 102096686 | CM007544.1:5573948-5574275 | CE | -0.5112 | 0.992 |
| male_pituitary_control vs male_pituitary_stress | 102086865 | CM007546.1:3072306-3072308 | AA | -0.42163 | 0.99 |
| male_pituitary_control vs male_pituitary_stress | 102098180 | CM007547.1:2986767-2986808 | CE | 0.39485 | 0.971 |
| male_pituitary_control vs male_pituitary_stress | 102085770 | CM007549.1:1449773-1450028 | TE | -0.54467 | 0.997 |
| male_pituitary_control vs male_pituitary_stress | 102085770 | CM007549.1:1450028-1450028 | TE | 0.53992 | 0.996 |
| male_pituitary_control vs male_pituitary_stress | 102085811 | CM007527.1:11396841-11396987 | TS | -0.51116 | 0.989 |
| male_pituitary_control vs male_pituitary_stress | 102085811 | CM007527.1:11396988-11397090 | TS | 0.50543 | 0.99 |
| male_pituitary_control vs male_pituitary_stress | 102088697 | CM007527.1:44153164-44153366 | CE | 0.43674 | 0.992 |
| male_pituitary_control vs male_pituitary_stress | 102084037 | CM007527.1:3683052-3683278 | RI | -0.41647 | 0.984 |
| male_pituitary_control vs male_pituitary_stress | 102084037 | CM007527.1:3683031-3683051 | CE | -0.53623 | 0.978 |
| male_pituitary_control vs male_pituitary_stress | 102084037 | CM007527.1:3682980-3683030 | AD | -0.44589 | 0.986 |
| male_pituitary_control vs male_pituitary_stress | 102097560 | CM007528.1:51294158-51294205 | CE | 0.51989 | 0.992 |
| male_pituitary_control vs male_pituitary_stress | 102093169 | CM007528.1:12059989-12060089 | AD | 0.48994 | 0.996 |
| male_pituitary_control vs male_pituitary_stress | 102089353 | CM007523.1:11197228-11202042 | TE | 0.50675 | 0.995 |
| male_pituitary_control vs male_pituitary_stress | 102089353 | CM007523.1:11202043-11202301 | TE | -0.51546 | 0.996 |
| male_pituitary_control vs male_pituitary_stress | 102086063 | CM007523.1:2303507-2303507 | AA | 0.59391 | 0.999 |
| male_pituitary_control vs male_pituitary_stress | 102085671 | CM007523.1:14102728-14102911 | CE | 0.13375 | 0.956 |
| male_pituitary_control vs male_pituitary_stress | 102093556 | CM007529.1:26120549-26121069 | CE | 0.4306 | 0.97 |
| male_pituitary_control vs male_pituitary_stress | 102088726 | CM007529.1:42052517-42052584 | CE | -0.37464 | 0.962 |
| male_pituitary_control vs male_pituitary_stress | 102088726 | CM007529.1:42053486-42053528 | CE | -0.38574 | 0.95 |
| male_pituitary_control vs male_pituitary_stress | 102088373 | CM007529.1:45681483-45681490 | AA | -0.53664 | 0.989 |
| male_pituitary_control vs male_pituitary_stress | 102085547 | CM007530.1:20143570-20143600 | AD | 0.59232 | 0.98 |
| male_pituitary_control vs male_pituitary_stress | 102095440 | CM007530.1:4859169-4859196 | CE | 0.33 | 0.972 |
| male_pituitary_control vs male_pituitary_stress | 102089916 | CM007530.1:2346700-2346879 | CE | -0.64196 | 0.994 |
| male_pituitary_control vs male_pituitary_stress | 102089159 | CM007530.1:34866749-34866856 | CE | 0.32091 | 0.957 |
| male_pituitary_control vs male_pituitary_stress | 102089159 | CM007530.1:34860715-34861026 | CE | 0.50216 | 0.993 |
| male_pituitary_control vs male_pituitary_stress | 102089159 | CM007530.1:34850429-34850536 | CE | 0.37248 | 0.988 |
| male_pituitary_control vs male_pituitary_stress | 102089159 | CM007530.1:34844697-34845008 | CE | 0.3019 | 0.989 |
| male_pituitary_control vs male_pituitary_stress | 102089159 | CM007530.1:34844457-34844561 | CE | 0.31689 | 0.994 |
| male_pituitary_control vs male_pituitary_stress | 102089159 | CM007530.1:34840315-34840419 | CE | 0.47635 | 0.997 |
| male_pituitary_control vs male_pituitary_stress | 102089159 | CM007530.1:34840138-34840242 | CE | 0.47952 | 0.999 |
| male_pituitary_control vs male_pituitary_stress | 102089159 | CM007530.1:34839549-34839656 | CE | 0.47535 | 0.994 |
| male_pituitary_control vs male_pituitary_stress | 102089159 | CM007530.1:34837329-34837433 | CE | 0.30295 | 0.951 |
| male_pituitary_control vs male_pituitary_stress | 102089159 | CM007530.1:34836488-34836592 | CE | 0.5599 | 0.998 |
| male_pituitary_control vs male_pituitary_stress | 102089159 | CM007530.1:34836133-34836240 | CE | 0.54341 | 0.995 |
| male_pituitary_control vs male_pituitary_stress | 102089159 | CM007530.1:34835716-34835823 | CE | 0.51628 | 0.994 |
| male_pituitary_control vs male_pituitary_stress | 102089159 | CM007530.1:34832509-34832613 | CE | 0.31358 | 1.0 |
| male_pituitary_control vs male_pituitary_stress | 102089159 | CM007530.1:34831989-34832093 | CE | 0.32657 | 0.999 |
| male_pituitary_control vs male_pituitary_stress | 102089159 | CM007530.1:34826867-34826971 | CE | -0.63701 | 1.0 |
| male_pituitary_control vs male_pituitary_stress | 102089159 | CM007530.1:34823507-34823611 | CE | 0.35288 | 1.0 |
| male_pituitary_control vs male_pituitary_stress | 102089159 | CM007530.1:34822750-34822854 | CE | 0.28319 | 0.954 |
| male_pituitary_control vs male_pituitary_stress | 102089159 | CM007530.1:34822537-34822641 | CE | 0.34283 | 1.0 |
| male_pituitary_control vs male_pituitary_stress | 102089159 | CM007530.1:34822156-34822260 | CE | 0.35092 | 1.0 |
| male_pituitary_control vs male_pituitary_stress | 102089159 | CM007530.1:34815508-34815612 | CE | 0.17775 | 0.97 |
| male_pituitary_control vs male_pituitary_stress | 102089159 | CM007530.1:34814297-34814401 | CE | 0.19844 | 0.986 |
| male_pituitary_control vs male_pituitary_stress | 102089159 | CM007530.1:34813327-34813437 | CE | 0.24485 | 0.967 |
| male_pituitary_control vs male_pituitary_stress | 102087010 | CM007530.1:229454-229499 | AA | 0.35306 | 0.978 |
| male_pituitary_control vs male_pituitary_stress | 102085426 | CM007531.1:12830087-12830218 | CE | -0.27381 | 0.974 |
| male_pituitary_control vs male_pituitary_stress | 102097170 | CM007531.1:5113393-5113393 | AA | -0.12407 | 0.962 |
| male_pituitary_control vs male_pituitary_stress | 102097170 | CM007531.1:5113393-5113444 | CE | -0.12337 | 0.956 |
| male_pituitary_control vs male_pituitary_stress | 102097170 | CM007531.1:5116712-5116734 | CE | 0.7731 | 1.0 |
| male_pituitary_control vs male_pituitary_stress | 102097170 | CM007531.1:5120417-5120620 | CE | 0.77087 | 1.0 |
| male_pituitary_control vs male_pituitary_stress | 102087434 | CM007531.1:13096456-13096527 | CE | -0.47433 | 0.986 |
| male_pituitary_control vs male_pituitary_stress | 102085253 | CM007532.1:8264914-8265180 | CE | 0.44488 | 0.953 |
| male_pituitary_control vs male_pituitary_stress | 102095309 | CM007532.1:11798582-11798641 | CE | -0.39419 | 0.977 |
| male_pituitary_control vs male_pituitary_stress | 102096408 | CM007532.1:5993051-5993922 | RI | 0.49044 | 0.97 |
| male_pituitary_control vs male_pituitary_stress | 102096292 | CM007532.1:12233400-12233471 | CE | -0.41644 | 0.967 |
| male_pituitary_control vs male_pituitary_stress | 102088721 | CM007532.1:10667177-10667209 | CE | 0.50436 | 0.981 |
| male_pituitary_control vs male_pituitary_stress | 102091932 | CM007533.1:7623743-7623805 | AA | -0.44158 | 0.969 |
| male_pituitary_control vs male_pituitary_stress | 102091459 | CM007533.1:12520509-12520583 | AA | 0.2729 | 0.953 |
| male_pituitary_control vs male_pituitary_stress | 102090828 | CM007533.1:21310068-21310220 | TS | -0.51722 | 0.995 |
| male_pituitary_control vs male_pituitary_stress | 102090828 | CM007533.1:21294695-21294785 | TS | 0.52175 | 0.991 |
| male_pituitary_control vs male_pituitary_stress | 102097088 | CM007524.1:36486458-36488088 | RI | 0.39228 | 0.982 |
| male_pituitary_control vs male_pituitary_stress | 102092000 | CM007524.1:5880351-5880373 | AD | -0.45015 | 0.969 |
| male_pituitary_control vs male_pituitary_stress | 103533213 | KV877988.1:1345467-1345587 | CE | 0.13014 | 0.971 |
| male_pituitary_control vs male_pituitary_stress | 102097670 | KV877989.1:142092-142241 | CE | 0.11289 | 0.951 |
| male_pituitary_control vs male_pituitary_stress | 102097670 | KV877989.1:139488-139575 | CE | 0.12083 | 0.968 |
| male_pituitary_control vs male_pituitary_stress | 102097670 | KV877989.1:139275-139392 | CE | 0.13531 | 0.969 |
| male_pituitary_control vs male_pituitary_stress | 102097670 | KV877989.1:138407-138662 | CE | 0.1138 | 0.987 |
| male_pituitary_control vs male_pituitary_stress | 102097670 | KV877989.1:135956-136345 | CE | 0.10632 | 0.991 |
| male_pituitary_control vs male_pituitary_stress | 102097670 | KV877989.1:130875-131000 | CE | 0.12856 | 0.999 |
| male_pituitary_control vs male_pituitary_stress | 102097670 | KV877989.1:130609-130779 | CE | 0.11198 | 0.996 |
| male_pituitary_control vs male_pituitary_stress | 102098553 | KV877989.1:17833-17978 | CE | 0.1356 | 0.973 |
| male_pituitary_control vs male_pituitary_stress | 102098190 | KV877989.1:47850-47974 | CE | 0.10527 | 0.955 |
| male_pituitary_control vs male_pituitary_stress | 102098732 | KV877989.1:187610-187755 | CE | 0.1206 | 0.983 |
| male_pituitary_control vs male_pituitary_stress | 102093886 | KV878003.1:844462-844473 | CE | 0.47483 | 0.989 |
| male_pituitary_control vs male_pituitary_stress | 102096431 | KV878011.1:13526057-13526489 | AA | 0.41711 | 0.968 |
| male_pituitary_control vs male_pituitary_stress | 102092721 | KV878015.1:907805-907876 | AD | -0.37974 | 0.962 |
| male_hypothalamus_control vs female_hypothalamus_control | 102095024 | CM007525.1:101515828-101516079 | CE | -0.43339 | 0.98 |
| male_hypothalamus_control vs female_hypothalamus_control | 102091116 | CM007525.1:59799905-59799908 | AA | 0.66264 | 1.0 |
| male_hypothalamus_control vs female_hypothalamus_control | 102094712 | CM007525.1:176927482-176927571 | CE | 0.29415 | 0.957 |
| male_hypothalamus_control vs female_hypothalamus_control | 102088390 | CM007525.1:195274189-195274457 | RI | 0.40718 | 0.964 |
| male_hypothalamus_control vs female_hypothalamus_control | 102086227 | CM007525.1:78705348-78705474 | CE | 0.56777 | 0.984 |
| male_hypothalamus_control vs female_hypothalamus_control | 102091304 | CM007525.1:175010335-175010439 | CE | 0.50824 | 0.984 |
| male_hypothalamus_control vs female_hypothalamus_control | 102090337 | CM007525.1:199316330-199316345 | CE | 0.61988 | 0.996 |
| male_hypothalamus_control vs female_hypothalamus_control | 102092636 | CM007525.1:175342557-175342642 | CE | -0.25103 | 0.951 |
| male_hypothalamus_control vs female_hypothalamus_control | 110358848 | CM007525.1:87987766-87987795 | CE | -0.51068 | 0.963 |
| male_hypothalamus_control vs female_hypothalamus_control | 106145910 | CM007525.1:117073402-117073489 | CE | -0.39999 | 0.984 |
| male_hypothalamus_control vs female_hypothalamus_control | 102094838 | CM007525.1:159833062-159833589 | RI | 0.40608 | 0.964 |
| male_hypothalamus_control vs female_hypothalamus_control | 102088757 | CM007534.1:6953541-6953555 | AD | 0.54725 | 0.961 |
| male_hypothalamus_control vs female_hypothalamus_control | 110365212 | CM007534.1:7099119-7100244 | RI | -0.47984 | 0.979 |
| male_hypothalamus_control vs female_hypothalamus_control | 102084238 | CM007534.1:4134843-4134877 | CE | 0.72653 | 0.998 |
| male_hypothalamus_control vs female_hypothalamus_control | 102089699 | CM007534.1:7691693-7691776 | CE | -0.73786 | 0.998 |
| male_hypothalamus_control vs female_hypothalamus_control | 102098594 | CM007535.1:3249227-3249240 | AD | 0.34274 | 0.965 |
| male_hypothalamus_control vs female_hypothalamus_control | 102083613 | CM007535.1:10894615-10894722 | RI | 0.74594 | 1.0 |
| male_hypothalamus_control vs female_hypothalamus_control | 102089642 | CM007537.1:17220600-17220683 | CE | 0.64959 | 1.0 |
| male_hypothalamus_control vs female_hypothalamus_control | 102085974 | CM007537.1:15687093-15687155 | CE | 0.329 | 0.987 |
| male_hypothalamus_control vs female_hypothalamus_control | 102094726 | CM007538.1:1084410-1084553 | CE | -0.30473 | 0.963 |
| male_hypothalamus_control vs female_hypothalamus_control | 102083933 | CM007540.1:8057457-8057484 | AA | 0.49453 | 0.984 |
| male_hypothalamus_control vs female_hypothalamus_control | 102086328 | CM007540.1:2034085-2034159 | CE | 0.44853 | 0.971 |
| male_hypothalamus_control vs female_hypothalamus_control | 102094263 | CM007540.1:6782370-6782377 | AD | -0.49763 | 0.99 |
| male_hypothalamus_control vs female_hypothalamus_control | 102091885 | CM007540.1:8597041-8597103 | CE | 0.56588 | 0.994 |
| male_hypothalamus_control vs female_hypothalamus_control | 102089640 | CM007541.1:8710694-8710828 | CE | 0.34637 | 0.959 |
| male_hypothalamus_control vs female_hypothalamus_control | 102094350 | CM007542.1:6987363-6987397 | AA | 0.70858 | 0.996 |
| male_hypothalamus_control vs female_hypothalamus_control | 102086394 | CM007542.1:5309164-5309188 | TS | -0.53865 | 0.995 |
| male_hypothalamus_control vs female_hypothalamus_control | 102086394 | CM007542.1:5309189-5309256 | TS | 0.53008 | 0.998 |
| male_hypothalamus_control vs female_hypothalamus_control | 102088057 | CM007526.1:26434984-26435200 | CE | -0.29462 | 0.971 |
| male_hypothalamus_control vs female_hypothalamus_control | 102086358 | CM007526.1:3942450-3942541 | CE | -0.24803 | 0.984 |
| male_hypothalamus_control vs female_hypothalamus_control | 102096825 | CM007526.1:45369874-45369948 | CE | -0.40407 | 0.975 |
| male_hypothalamus_control vs female_hypothalamus_control | 102094422 | CM007526.1:48257532-48258618 | RI | -0.40803 | 0.963 |
| male_hypothalamus_control vs female_hypothalamus_control | 102092093 | CM007526.1:8867772-8867880 | CE | 0.71178 | 1.0 |
| male_hypothalamus_control vs female_hypothalamus_control | 102095911 | CM007526.1:44968041-44968175 | CE | 0.30194 | 0.984 |
| male_hypothalamus_control vs female_hypothalamus_control | 102095911 | CM007526.1:44969622-44969723 | CE | 0.32354 | 0.987 |
| male_hypothalamus_control vs female_hypothalamus_control | 102092335 | CM007526.1:113549045-113549066 | AA | -0.50413 | 0.969 |
| male_hypothalamus_control vs female_hypothalamus_control | 102085412 | CM007526.1:131182551-131182604 | CE | -0.56225 | 0.993 |
| male_hypothalamus_control vs female_hypothalamus_control | 102085412 | CM007526.1:131177634-131177687 | CE | -0.55279 | 0.994 |
| male_hypothalamus_control vs female_hypothalamus_control | 102085412 | CM007526.1:131163713-131163766 | CE | -0.47571 | 0.988 |
| male_hypothalamus_control vs female_hypothalamus_control | 102085412 | CM007526.1:131162732-131162785 | CE | -0.49841 | 0.988 |
| male_hypothalamus_control vs female_hypothalamus_control | 102096169 | CM007526.1:88160982-88161088 | CE | -0.47818 | 0.988 |
| male_hypothalamus_control vs female_hypothalamus_control | 102096169 | CM007526.1:88163892-88163942 | CE | -0.41756 | 0.97 |
| male_hypothalamus_control vs female_hypothalamus_control | 102084570 | CM007526.1:72549875-72549895 | AD | -0.37456 | 0.966 |
| male_hypothalamus_control vs female_hypothalamus_control | 102084570 | CM007526.1:72550030-72552225 | RI | 0.46026 | 0.979 |
| male_hypothalamus_control vs female_hypothalamus_control | 102084352 | CM007526.1:22105709-22105737 | RI | -0.76625 | 0.998 |
| male_hypothalamus_control vs female_hypothalamus_control | 102091635 | CM007544.1:5495979-5496058 | CE | 0.71049 | 1.0 |
| male_hypothalamus_control vs female_hypothalamus_control | 102087006 | CM007544.1:310883-310896 | AD | -0.7535 | 0.998 |
| male_hypothalamus_control vs female_hypothalamus_control | 102087608 | CM007544.1:1560247-1565077 | RI | 0.65638 | 0.987 |
| male_hypothalamus_control vs female_hypothalamus_control | 102087608 | CM007544.1:1565078-1565081 | AA | 0.67989 | 0.987 |
| male_hypothalamus_control vs female_hypothalamus_control | 102085942 | CM007546.1:2120078-2121037 | AD | -0.50712 | 0.982 |
| male_hypothalamus_control vs female_hypothalamus_control | 102095747 | CM007546.1:652895-652899 | AD | -0.74812 | 0.998 |
| male_hypothalamus_control vs female_hypothalamus_control | 102097419 | CM007546.1:5605760-5607193 | TE | -0.53245 | 0.998 |
| male_hypothalamus_control vs female_hypothalamus_control | 102097419 | CM007546.1:5607194-5607196 | TE | 0.52907 | 0.999 |
| male_hypothalamus_control vs female_hypothalamus_control | 102085822 | CM007549.1:1274860-1275109 | CE | -0.28214 | 0.963 |
| male_hypothalamus_control vs female_hypothalamus_control | 102086160 | CM007551.1:864538-864942 | RI | -0.50197 | 0.97 |
| male_hypothalamus_control vs female_hypothalamus_control | 102086160 | CM007551.1:864448-864537 | CE | -0.52275 | 0.985 |
| male_hypothalamus_control vs female_hypothalamus_control | 102087405 | CM007551.1:1036110-1036172 | CE | 0.41668 | 0.976 |
| male_hypothalamus_control vs female_hypothalamus_control | 102094122 | CM007527.1:89760753-89760891 | CE | -0.34117 | 0.954 |
| male_hypothalamus_control vs female_hypothalamus_control | 102091617 | CM007527.1:26758381-26758399 | AD | 0.63781 | 0.993 |
| male_hypothalamus_control vs female_hypothalamus_control | 102093328 | CM007527.1:111265894-111266028 | CE | -0.35732 | 0.957 |
| male_hypothalamus_control vs female_hypothalamus_control | 102092123 | CM007527.1:30800224-30800290 | CE | -0.51913 | 0.992 |
| male_hypothalamus_control vs female_hypothalamus_control | 102089252 | CM007527.1:24548935-24548935 | AD | 0.72797 | 1.0 |
| male_hypothalamus_control vs female_hypothalamus_control | 102088088 | CM007527.1:1036146-1036448 | RI | -0.66685 | 0.994 |
| male_hypothalamus_control vs female_hypothalamus_control | 102095782 | CM007527.1:34000615-34000700 | CE | 0.40579 | 0.973 |
| male_hypothalamus_control vs female_hypothalamus_control | 102085483 | CM007528.1:24724892-24724938 | AA | 0.4356 | 0.987 |
| male_hypothalamus_control vs female_hypothalamus_control | 102083532 | CM007523.1:6960901-6960912 | AA | -0.489 | 0.988 |
| male_hypothalamus_control vs female_hypothalamus_control | 102096298 | CM007523.1:12460006-12460397 | AA | 0.38017 | 0.952 |
| male_hypothalamus_control vs female_hypothalamus_control | 102086804 | CM007523.1:2364011-2364127 | AA | 0.42843 | 0.959 |
| male_hypothalamus_control vs female_hypothalamus_control | 102085132 | CM007529.1:8381675-8381776 | CE | -0.31083 | 0.951 |
| male_hypothalamus_control vs female_hypothalamus_control | 102088314 | CM007529.1:41945866-41945997 | AA | 0.6415 | 0.999 |
| male_hypothalamus_control vs female_hypothalamus_control | 102090171 | CM007530.1:10561460-10561863 | RI | -0.35056 | 0.971 |
| male_hypothalamus_control vs female_hypothalamus_control | 102095997 | CM007530.1:4955797-4956018 | TE | 0.53137 | 0.996 |
| male_hypothalamus_control vs female_hypothalamus_control | 102095997 | CM007530.1:4956019-4956072 | TE | -0.52348 | 0.991 |
| male_hypothalamus_control vs female_hypothalamus_control | 102093658 | CM007530.1:2534040-2534057 | CE | 0.66402 | 0.978 |
| male_hypothalamus_control vs female_hypothalamus_control | 102093658 | CM007530.1:2537921-2537968 | CE | 0.46005 | 0.953 |
| male_hypothalamus_control vs female_hypothalamus_control | 102093658 | CM007530.1:2544887-2544934 | CE | 0.4114 | 0.979 |
| male_hypothalamus_control vs female_hypothalamus_control | 102094674 | CM007531.1:20476680-20476836 | CE | -0.40863 | 0.976 |
| male_hypothalamus_control vs female_hypothalamus_control | 102090408 | CM007531.1:20218166-20218493 | RI | 0.42002 | 0.972 |
| male_hypothalamus_control vs female_hypothalamus_control | 102097377 | CM007532.1:13292365-13292447 | RI | -0.57185 | 0.982 |
| male_hypothalamus_control vs female_hypothalamus_control | 102084643 | CM007532.1:22281571-22281820 | AF | -0.51739 | 0.981 |
| male_hypothalamus_control vs female_hypothalamus_control | 102089233 | CM007532.1:10776087-10776173 | AL | 0.31371 | 0.955 |
| male_hypothalamus_control vs female_hypothalamus_control | 102083979 | CM007533.1:10137747-10137770 | RI | -0.45358 | 0.954 |
| male_hypothalamus_control vs female_hypothalamus_control | 102098416 | CM007533.1:14145644-14145675 | CE | -0.45748 | 0.977 |
| male_hypothalamus_control vs female_hypothalamus_control | 102097687 | CM007524.1:31423387-31423463 | AD | 0.58971 | 0.986 |
| male_hypothalamus_control vs female_hypothalamus_control | 102093302 | CM007524.1:19620105-19621355 | RI | 0.47413 | 0.977 |
| male_hypothalamus_control vs female_hypothalamus_control | 102097097 | CM007524.1:23498966-23499560 | RI | -0.2604 | 0.952 |
| male_hypothalamus_control vs female_hypothalamus_control | 102090375 | CM007524.1:15177231-15177317 | RI | -0.49647 | 0.983 |
| male_hypothalamus_control vs female_hypothalamus_control | 102091397 | CM007524.1:21070644-21070719 | CE | 0.35344 | 0.989 |
| male_hypothalamus_control vs female_hypothalamus_control | 102092000 | CM007524.1:5880194-5880350 | RI | -0.54817 | 0.997 |
| male_hypothalamus_control vs female_hypothalamus_control | 102092554 | CM007524.1:37436157-37436207 | CE | -0.44584 | 0.965 |
| male_hypothalamus_control vs female_hypothalamus_control | 106145966 | KV877987.1:822729-822820 | AA | 0.3349 | 0.964 |
| male_hypothalamus_control vs female_hypothalamus_control | 102086537 | KV877990.1:1467043-1467048 | CE | 0.76599 | 1.0 |
| male_hypothalamus_control vs female_hypothalamus_control | 102089681 | KV877996.1:136362-136625 | RI | -0.50737 | 0.987 |
| male_hypothalamus_control vs female_hypothalamus_control | 102089748 | KV877999.1:3858029-3858116 | CE | 0.43926 | 0.977 |
| male_hypothalamus_control vs female_hypothalamus_control | 102085598 | KV877999.1:13825766-13825812 | CE | -0.60514 | 0.996 |
| male_hypothalamus_control vs female_hypothalamus_control | 102097653 | KV877999.1:520737-522313 | RI | 0.56243 | 0.998 |
| male_hypothalamus_control vs female_hypothalamus_control | 102092021 | KV878003.1:357829-358493 | TE | -0.4827 | 0.978 |
| male_hypothalamus_control vs female_hypothalamus_control | 102092021 | KV878003.1:358494-358529 | TE | 0.48532 | 0.979 |
| male_hypothalamus_control vs female_hypothalamus_control | 102085700 | KV878003.1:6460487-6460519 | CE | 0.5216 | 0.992 |
| male_hypothalamus_stress vs female_hypothalamus_stress | 102085623 | CM007525.1:192046586-192046798 | CE | 0.67424 | 0.998 |
| male_hypothalamus_stress vs female_hypothalamus_stress | 102086227 | CM007525.1:78702758-78702839 | CE | -0.35223 | 0.961 |
| male_hypothalamus_stress vs female_hypothalamus_stress | 102092428 | CM007525.1:113027886-113027966 | CE | 0.43854 | 0.981 |
| male_hypothalamus_stress vs female_hypothalamus_stress | 102097217 | CM007525.1:760081-760131 | AD | -0.23974 | 0.972 |
| male_hypothalamus_stress vs female_hypothalamus_stress | 102097217 | CM007525.1:760132-760158 | AD | -0.25323 | 0.969 |
| male_hypothalamus_stress vs female_hypothalamus_stress | 102084196 | CM007525.1:35955741-35955818 | AD | 0.49096 | 0.998 |
| male_hypothalamus_stress vs female_hypothalamus_stress | 102096853 | CM007525.1:85101890-85102225 | CE | 0.23723 | 0.961 |
| male_hypothalamus_stress vs female_hypothalamus_stress | 102097356 | CM007534.1:2965516-2965652 | CE | 0.36634 | 0.968 |
| male_hypothalamus_stress vs female_hypothalamus_stress | 102088757 | CM007534.1:6953541-6953555 | AD | 0.65399 | 1.0 |
| male_hypothalamus_stress vs female_hypothalamus_stress | 102090369 | CM007534.1:18533519-18533674 | AA | -0.32683 | 0.98 |
| male_hypothalamus_stress vs female_hypothalamus_stress | 102084238 | CM007534.1:4134878-4135067 | RI | 0.42531 | 0.976 |
| male_hypothalamus_stress vs female_hypothalamus_stress | 102098932 | CM007535.1:15101140-15101307 | RI | -0.42555 | 0.964 |
| male_hypothalamus_stress vs female_hypothalamus_stress | 102090470 | CM007535.1:1499038-1499042 | AD | -0.4291 | 0.98 |
| male_hypothalamus_stress vs female_hypothalamus_stress | 102098187 | CM007536.1:9508460-9508571 | RI | -0.3618 | 0.952 |
| male_hypothalamus_stress vs female_hypothalamus_stress | 102091392 | CM007536.1:4276795-4276806 | AD | -0.43305 | 0.977 |
| male_hypothalamus_stress vs female_hypothalamus_stress | 102090698 | CM007539.1:5097763-5097894 | CE | -0.45768 | 0.963 |
| male_hypothalamus_stress vs female_hypothalamus_stress | 102097815 | CM007541.1:4972559-4972702 | CE | 0.33072 | 0.95 |
| male_hypothalamus_stress vs female_hypothalamus_stress | 102097778 | CM007526.1:31901754-31901865 | CE | -0.4113 | 0.98 |
| male_hypothalamus_stress vs female_hypothalamus_stress | 102096169 | CM007526.1:88156608-88156655 | AA | -0.32605 | 0.952 |
| male_hypothalamus_stress vs female_hypothalamus_stress | 102090771 | CM007526.1:24607573-24607678 | AD | 0.42509 | 0.998 |
| male_hypothalamus_stress vs female_hypothalamus_stress | 102083948 | CM007526.1:34529896-34530006 | CE | 0.38681 | 0.99 |
| male_hypothalamus_stress vs female_hypothalamus_stress | 102088288 | CM007544.1:99007-99009 | AA | -0.29996 | 0.979 |
| male_hypothalamus_stress vs female_hypothalamus_stress | 102096327 | CM007549.1:2329549-2329579 | AA | -0.46098 | 0.96 |
| male_hypothalamus_stress vs female_hypothalamus_stress | 102088892 | CM007527.1:17762373-17764109 | AA | 0.31433 | 0.957 |
| male_hypothalamus_stress vs female_hypothalamus_stress | 102092123 | CM007527.1:30800224-30800290 | CE | 0.32851 | 0.952 |
| male_hypothalamus_stress vs female_hypothalamus_stress | 102091530 | CM007528.1:2809740-2810313 | AD | 0.35981 | 0.968 |
| male_hypothalamus_stress vs female_hypothalamus_stress | 102088785 | CM007528.1:21459832-21459969 | TS | -0.54156 | 0.999 |
| male_hypothalamus_stress vs female_hypothalamus_stress | 102088785 | CM007528.1:21459970-21460231 | TS | 0.54718 | 0.999 |
| male_hypothalamus_stress vs female_hypothalamus_stress | 104833389 | CM007528.1:27521271-27521343 | RI | -0.3886 | 0.965 |
| male_hypothalamus_stress vs female_hypothalamus_stress | 102092393 | CM007528.1:14776829-14776970 | CE | -0.45219 | 0.996 |
| male_hypothalamus_stress vs female_hypothalamus_stress | 102092393 | CM007528.1:14777275-14777362 | CE | -0.46038 | 0.995 |
| male_hypothalamus_stress vs female_hypothalamus_stress | 102092393 | CM007528.1:14778659-14778919 | CE | -0.50891 | 0.996 |
| male_hypothalamus_stress vs female_hypothalamus_stress | 102092393 | CM007528.1:14779229-14779364 | CE | -0.41533 | 0.989 |
| male_hypothalamus_stress vs female_hypothalamus_stress | 102092393 | CM007528.1:14791592-14791679 | CE | -0.41604 | 0.987 |
| male_hypothalamus_stress vs female_hypothalamus_stress | 102092393 | CM007528.1:14791795-14792014 | CE | -0.54779 | 0.998 |
| male_hypothalamus_stress vs female_hypothalamus_stress | 102093831 | CM007528.1:16400722-16401201 | RI | -0.32069 | 0.955 |
| male_hypothalamus_stress vs female_hypothalamus_stress | 102085663 | CM007529.1:9262974-9263061 | CE | -0.37851 | 0.967 |
| male_hypothalamus_stress vs female_hypothalamus_stress | 102084706 | CM007529.1:26858677-26858723 | AA | 0.38427 | 0.954 |
| male_hypothalamus_stress vs female_hypothalamus_stress | 102092530 | CM007529.1:25618246-25618311 | CE | 0.45094 | 0.984 |
| male_hypothalamus_stress vs female_hypothalamus_stress | 102096698 | CM007529.1:2458370-2459599 | RI | -0.33639 | 0.964 |
| male_hypothalamus_stress vs female_hypothalamus_stress | 102087151 | CM007529.1:23927670-23927836 | CE | -0.61385 | 0.995 |
| male_hypothalamus_stress vs female_hypothalamus_stress | 102085081 | CM007529.1:27118401-27118574 | CE | -0.30136 | 0.969 |
| male_hypothalamus_stress vs female_hypothalamus_stress | 102091175 | CM007530.1:18592275-18592369 | CE | 0.39734 | 0.954 |
| male_hypothalamus_stress vs female_hypothalamus_stress | 102096295 | CM007531.1:12627271-12627309 | CE | 0.36197 | 0.956 |
| male_hypothalamus_stress vs female_hypothalamus_stress | 102084230 | CM007531.1:24032737-24032858 | CE | -0.59074 | 0.996 |
| male_hypothalamus_stress vs female_hypothalamus_stress | 102097170 | CM007531.1:5116712-5116734 | CE | -0.75734 | 1.0 |
| male_hypothalamus_stress vs female_hypothalamus_stress | 102097170 | CM007531.1:5120417-5120620 | CE | -0.46734 | 0.993 |
| male_hypothalamus_stress vs female_hypothalamus_stress | 102095309 | CM007532.1:11798582-11798641 | CE | -0.36161 | 0.958 |
| male_hypothalamus_stress vs female_hypothalamus_stress | 102088300 | CM007532.1:16915632-16915675 | CE | -0.41985 | 0.961 |
| male_hypothalamus_stress vs female_hypothalamus_stress | 102086573 | CM007532.1:8639003-8639047 | AA | -0.46056 | 0.976 |
| male_hypothalamus_stress vs female_hypothalamus_stress | 103901448 | CM007533.1:12657296-12657329 | AD | 0.45164 | 0.993 |
| male_hypothalamus_stress vs female_hypothalamus_stress | 102095462 | KV877988.1:206232-206282 | CE | -0.36206 | 0.966 |
| male_hypothalamus_stress vs female_hypothalamus_stress | 102088881 | KV877991.1:1157005-1157075 | CE | -0.30539 | 0.957 |
| male_hypothalamus_stress vs female_hypothalamus_stress | 102085598 | KV877999.1:13823426-13823914 | RI | -0.36622 | 0.953 |
| male_hypothalamus_stress vs female_hypothalamus_stress | 102087631 | KV878000.1:6887034-6888032 | AD | 0.41325 | 0.969 |
| male_hypothalamus_stress vs female_hypothalamus_stress | 102094305 | KV878001.1:959028-959191 | CE | 0.39853 | 0.97 |
| male_hypothalamus_stress vs female_hypothalamus_stress | 102094490 | KV878004.1:3076284-3076301 | AA | 0.48264 | 0.978 |
| male_hypothalamus_stress vs female_hypothalamus_stress | 102094490 | KV878004.1:3082306-3082345 | AD | -0.46562 | 0.977 |
| male_hypothalamus_stress vs female_hypothalamus_stress | 102094490 | KV878004.1:3093702-3094558 | AD | -0.48272 | 0.989 |
| male_pituitary_control vs female_pituitary_control | 102097908 | CM007525.1:52795175-52795266 | AD | 0.34691 | 0.985 |
| male_pituitary_control vs female_pituitary_control | 102096781 | CM007525.1:68550332-68550563 | CE | -0.11511 | 0.971 |
| male_pituitary_control vs female_pituitary_control | 102091538 | CM007525.1:67148785-67148975 | CE | 0.78501 | 1.0 |
| male_pituitary_control vs female_pituitary_control | 102088097 | CM007525.1:40351219-40351246 | AA | 0.47799 | 0.994 |
| male_pituitary_control vs female_pituitary_control | 102094712 | CM007525.1:176931742-176932581 | AD | -0.43902 | 0.961 |
| male_pituitary_control vs female_pituitary_control | 102086210 | CM007525.1:175168373-175168393 | AA | 0.78108 | 1.0 |
| male_pituitary_control vs female_pituitary_control | 102086221 | CM007525.1:138817345-138817501 | CE | -0.4729 | 0.993 |
| male_pituitary_control vs female_pituitary_control | 102096569 | CM007525.1:133193502-133193621 | AA | 0.43625 | 0.988 |
| male_pituitary_control vs female_pituitary_control | 102085809 | CM007525.1:134954523-134954597 | CE | -0.64042 | 0.996 |
| male_pituitary_control vs female_pituitary_control | 102087423 | CM007525.1:177680740-177680780 | CE | 0.28838 | 0.953 |
| male_pituitary_control vs female_pituitary_control | 102090452 | CM007525.1:48648978-48649147 | CE | -0.52466 | 0.997 |
| male_pituitary_control vs female_pituitary_control | 102094693 | CM007525.1:192532244-192532279 | CE | 0.24499 | 0.952 |
| male_pituitary_control vs female_pituitary_control | 102090098 | CM007525.1:68698940-68698993 | CE | 0.58009 | 0.999 |
| male_pituitary_control vs female_pituitary_control | 102094583 | CM007525.1:89906281-89908156 | AA | 0.36783 | 0.951 |
| male_pituitary_control vs female_pituitary_control | 102087259 | CM007534.1:8642708-8642755 | AA | 0.33559 | 0.959 |
| male_pituitary_control vs female_pituitary_control | 102084780 | CM007535.1:4674605-4674624 | RI | 0.73956 | 0.999 |
| male_pituitary_control vs female_pituitary_control | 110362309 | CM007535.1:20721788-20721899 | CE | -0.45515 | 0.969 |
| male_pituitary_control vs female_pituitary_control | 110362309 | CM007535.1:20721545-20721787 | AD | -0.43651 | 0.951 |
| male_pituitary_control vs female_pituitary_control | 102097851 | CM007535.1:16374024-16374110 | CE | -0.41907 | 0.975 |
| male_pituitary_control vs female_pituitary_control | 102084901 | CM007535.1:11428058-11428149 | CE | 0.364 | 0.962 |
| male_pituitary_control vs female_pituitary_control | 102092413 | CM007537.1:10275951-10275996 | AD | 0.37455 | 0.951 |
| male_pituitary_control vs female_pituitary_control | 102085568 | CM007537.1:15596933-15597309 | RI | 0.49456 | 0.981 |
| male_pituitary_control vs female_pituitary_control | 102085569 | CM007537.1:4558343-4558871 | CE | -0.1397 | 0.982 |
| male_pituitary_control vs female_pituitary_control | 102085569 | CM007537.1:4564041-4564201 | CE | -0.13974 | 0.992 |
| male_pituitary_control vs female_pituitary_control | 102096732 | CM007537.1:8487982-8488021 | AA | 0.30732 | 0.956 |
| male_pituitary_control vs female_pituitary_control | 102085974 | CM007537.1:15682059-15682122 | CE | -0.71994 | 1.0 |
| male_pituitary_control vs female_pituitary_control | 102088507 | CM007538.1:2693177-2694450 | CE | -0.53879 | 0.968 |
| male_pituitary_control vs female_pituitary_control | 102089633 | CM007538.1:2784454-2784708 | CE | -0.35296 | 0.956 |
| male_pituitary_control vs female_pituitary_control | 102084030 | CM007538.1:2192807-2192897 | AA | -0.41548 | 0.973 |
| male_pituitary_control vs female_pituitary_control | 102093801 | CM007538.1:4690087-4690249 | CE | -0.31053 | 0.956 |
| male_pituitary_control vs female_pituitary_control | 102088530 | CM007540.1:4869020-4869196 | CE | 0.3886 | 0.964 |
| male_pituitary_control vs female_pituitary_control | 102098765 | CM007541.1:4756374-4756430 | CE | 0.31495 | 0.952 |
| male_pituitary_control vs female_pituitary_control | 102084611 | CM007541.1:6641504-6641600 | CE | -0.11515 | 0.978 |
| male_pituitary_control vs female_pituitary_control | 102084611 | CM007541.1:6642967-6643043 | CE | -0.12033 | 0.98 |
| male_pituitary_control vs female_pituitary_control | 102084611 | CM007541.1:6644389-6644568 | CE | -0.14449 | 0.984 |
| male_pituitary_control vs female_pituitary_control | 102084611 | CM007541.1:6645044-6645241 | CE | -0.13908 | 0.986 |
| male_pituitary_control vs female_pituitary_control | 102084176 | CM007541.1:8727750-8727753 | AD | 0.79458 | 1.0 |
| male_pituitary_control vs female_pituitary_control | 102084176 | CM007541.1:8727694-8727749 | AD | 0.73535 | 1.0 |
| male_pituitary_control vs female_pituitary_control | 102090571 | CM007542.1:7629850-7629989 | AA | -0.36517 | 0.964 |
| male_pituitary_control vs female_pituitary_control | 110359246 | CM007542.1:8600377-8600483 | CE | -0.33123 | 0.978 |
| male_pituitary_control vs female_pituitary_control | 102090819 | CM007526.1:114747817-114747819 | TS | -0.45802 | 0.991 |
| male_pituitary_control vs female_pituitary_control | 102090819 | CM007526.1:114746790-114747816 | TS | 0.44961 | 0.993 |
| male_pituitary_control vs female_pituitary_control | 102090423 | CM007526.1:89515738-89515818 | CE | -0.13407 | 0.967 |
| male_pituitary_control vs female_pituitary_control | 102090702 | CM007526.1:110587018-110587130 | CE | -0.11803 | 0.954 |
| male_pituitary_control vs female_pituitary_control | 102087186 | CM007526.1:32819960-32820040 | CE | -0.11396 | 0.965 |
| male_pituitary_control vs female_pituitary_control | 102097223 | CM007526.1:98813240-98813245 | AA | -0.4327 | 0.958 |
| male_pituitary_control vs female_pituitary_control | 102098693 | CM007543.1:7917897-7918037 | CE | -0.34957 | 0.999 |
| male_pituitary_control vs female_pituitary_control | 102098693 | CM007543.1:7920435-7920470 | CE | -0.12075 | 0.961 |
| male_pituitary_control vs female_pituitary_control | 102088856 | CM007543.1:1686355-1686491 | CE | 0.14123 | 1.0 |
| male_pituitary_control vs female_pituitary_control | 102091635 | CM007544.1:5490894-5490966 | CE | -0.30944 | 0.968 |
| male_pituitary_control vs female_pituitary_control | 102091635 | CM007544.1:5490756-5490817 | CE | -0.31046 | 0.97 |
| male_pituitary_control vs female_pituitary_control | 102096686 | CM007544.1:5573948-5574275 | CE | -0.50863 | 0.993 |
| male_pituitary_control vs female_pituitary_control | 102091662 | CM007544.1:136716-136771 | AD | 0.64241 | 0.999 |
| male_pituitary_control vs female_pituitary_control | 102088149 | CM007546.1:2919903-2920073 | CE | 0.33613 | 0.956 |
| male_pituitary_control vs female_pituitary_control | 102097634 | CM007547.1:3777431-3777553 | CE | -0.10031 | 0.965 |
| male_pituitary_control vs female_pituitary_control | 102086187 | CM007550.1:4100279-4100336 | CE | -0.39003 | 0.972 |
| male_pituitary_control vs female_pituitary_control | 102089384 | CM007550.1:459460-459520 | CE | -0.12582 | 0.988 |
| male_pituitary_control vs female_pituitary_control | 102089384 | CM007550.1:457543-457763 | CE | -0.10765 | 0.971 |
| male_pituitary_control vs female_pituitary_control | 102089384 | CM007550.1:457314-457357 | CE | -0.10971 | 0.982 |
| male_pituitary_control vs female_pituitary_control | 102091934 | CM007527.1:88128135-88128239 | AD | -0.48456 | 0.994 |
| male_pituitary_control vs female_pituitary_control | 102088697 | CM007527.1:44153164-44153366 | CE | 0.24417 | 0.956 |
| male_pituitary_control vs female_pituitary_control | 102084408 | CM007527.1:80076251-80076307 | CE | -0.50936 | 0.988 |
| male_pituitary_control vs female_pituitary_control | 102084408 | CM007527.1:80098138-80098404 | CE | -0.65437 | 1.0 |
| male_pituitary_control vs female_pituitary_control | 102084408 | CM007527.1:80099725-80099917 | CE | -0.6147 | 1.0 |
| male_pituitary_control vs female_pituitary_control | 102096543 | CM007527.1:24575901-24578899 | TE | -0.47968 | 0.979 |
| male_pituitary_control vs female_pituitary_control | 102096543 | CM007527.1:24578900-24578900 | TE | 0.47898 | 0.978 |
| male_pituitary_control vs female_pituitary_control | 102090468 | CM007527.1:26938524-26938664 | CE | 0.56135 | 0.998 |
| male_pituitary_control vs female_pituitary_control | 102093700 | CM007527.1:106273696-106273889 | RI | 0.29788 | 0.963 |
| male_pituitary_control vs female_pituitary_control | 102093328 | CM007527.1:111265894-111266028 | CE | -0.33419 | 0.978 |
| male_pituitary_control vs female_pituitary_control | 102089442 | CM007527.1:22599159-22599254 | CE | -0.2966 | 0.956 |
| male_pituitary_control vs female_pituitary_control | 102089442 | CM007527.1:22595619-22595693 | CE | -0.28206 | 0.958 |
| male_pituitary_control vs female_pituitary_control | 102089513 | CM007527.1:12233496-12233627 | CE | 0.41794 | 0.992 |
| male_pituitary_control vs female_pituitary_control | 102090335 | CM007528.1:62114150-62114562 | RI | -0.5617 | 0.989 |
| male_pituitary_control vs female_pituitary_control | 102093169 | CM007528.1:12059989-12060089 | AD | 0.40755 | 0.973 |
| male_pituitary_control vs female_pituitary_control | 102093169 | CM007528.1:12060090-12060518 | RI | 0.59132 | 1.0 |
| male_pituitary_control vs female_pituitary_control | 104836833 | CM007528.1:2787093-2787305 | CE | -0.46302 | 0.952 |
| male_pituitary_control vs female_pituitary_control | 102096298 | CM007523.1:12460006-12460397 | AA | 0.38445 | 0.951 |
| male_pituitary_control vs female_pituitary_control | 102089772 | CM007523.1:3325763-3325794 | AF | 0.10067 | 0.997 |
| male_pituitary_control vs female_pituitary_control | 102089772 | CM007523.1:3327517-3327693 | AD | -0.12188 | 0.993 |
| male_pituitary_control vs female_pituitary_control | 102089772 | CM007523.1:3328010-3328150 | AA | 0.12688 | 0.99 |
| male_pituitary_control vs female_pituitary_control | 102085525 | CM007523.1:9952250-9952429 | AA | -0.36388 | 0.954 |
| male_pituitary_control vs female_pituitary_control | 102088965 | CM007529.1:7291926-7292038 | AL | 0.32871 | 0.986 |
| male_pituitary_control vs female_pituitary_control | 102090397 | CM007529.1:45670309-45670354 | CE | -0.48168 | 0.994 |
| male_pituitary_control vs female_pituitary_control | 102090563 | CM007529.1:15333100-15333189 | CE | 0.14099 | 0.997 |
| male_pituitary_control vs female_pituitary_control | 102096698 | CM007529.1:2380162-2380270 | CE | 0.37181 | 0.983 |
| male_pituitary_control vs female_pituitary_control | 102095199 | CM007529.1:24775470-24775552 | AA | -0.38345 | 0.95 |
| male_pituitary_control vs female_pituitary_control | 102091177 | CM007529.1:15243580-15243696 | CE | 0.14751 | 1.0 |
| male_pituitary_control vs female_pituitary_control | 102091177 | CM007529.1:15243158-15243235 | CE | 0.12961 | 0.999 |
| male_pituitary_control vs female_pituitary_control | 102091177 | CM007529.1:15241556-15241646 | CE | 0.13778 | 0.999 |
| male_pituitary_control vs female_pituitary_control | 110365155 | CM007529.1:1982810-1982832 | RI | 0.74726 | 1.0 |
| male_pituitary_control vs female_pituitary_control | 110365155 | CM007529.1:1982803-1982809 | AA | 0.37465 | 0.974 |
| male_pituitary_control vs female_pituitary_control | 102088599 | CM007529.1:7656957-7658534 | RI | 0.42115 | 0.998 |
| male_pituitary_control vs female_pituitary_control | 102091060 | CM007530.1:15569048-15569068 | CE | -0.46956 | 0.975 |
| male_pituitary_control vs female_pituitary_control | 102085547 | CM007530.1:20143570-20143600 | AD | 0.51393 | 0.993 |
| male_pituitary_control vs female_pituitary_control | 102090171 | CM007530.1:10561460-10561863 | RI | 0.53066 | 0.969 |
| male_pituitary_control vs female_pituitary_control | 102096534 | CM007530.1:5308437-5308539 | CE | -0.71675 | 1.0 |
| male_pituitary_control vs female_pituitary_control | 102089159 | CM007530.1:34826867-34826971 | CE | -0.85193 | 1.0 |
| male_pituitary_control vs female_pituitary_control | 102089159 | CM007530.1:34814297-34814401 | CE | 0.31066 | 0.999 |
| male_pituitary_control vs female_pituitary_control | 102089159 | CM007530.1:34813327-34813437 | CE | 0.48386 | 1.0 |
| male_pituitary_control vs female_pituitary_control | 102097566 | CM007531.1:8007731-8008415 | AD | -0.51131 | 0.992 |
| male_pituitary_control vs female_pituitary_control | 102098814 | CM007531.1:11493236-11494732 | CE | -0.50627 | 0.993 |
| male_pituitary_control vs female_pituitary_control | 102087434 | CM007531.1:13096456-13096527 | CE | -0.40891 | 0.968 |
| male_pituitary_control vs female_pituitary_control | 102083465 | CM007531.1:2972581-2972606 | AD | 0.24825 | 0.959 |
| male_pituitary_control vs female_pituitary_control | 102097377 | CM007532.1:13293089-13293274 | CE | 0.3902 | 0.966 |
| male_pituitary_control vs female_pituitary_control | 102095309 | CM007532.1:11798582-11798641 | CE | -0.36543 | 0.969 |
| male_pituitary_control vs female_pituitary_control | 102087986 | CM007532.1:10583861-10585088 | RI | 0.48401 | 0.977 |
| male_pituitary_control vs female_pituitary_control | 102096292 | CM007532.1:12233400-12233471 | CE | -0.55527 | 0.994 |
| male_pituitary_control vs female_pituitary_control | 102088241 | CM007532.1:25716365-25716535 | CE | -0.13252 | 0.969 |
| male_pituitary_control vs female_pituitary_control | 102098436 | CM007533.1:22069434-22070471 | AL | 0.38947 | 0.965 |
| male_pituitary_control vs female_pituitary_control | 102091563 | CM007533.1:7893646-7895204 | RI | 0.39079 | 0.958 |
| male_pituitary_control vs female_pituitary_control | 102085587 | CM007524.1:25512484-25512510 | AD | 0.48274 | 0.986 |
| male_pituitary_control vs female_pituitary_control | 106145967 | KV877987.1:599280-599321 | CE | -0.42434 | 0.971 |
| male_pituitary_control vs female_pituitary_control | 102092371 | KV877988.1:1243615-1243713 | CE | -0.43764 | 0.988 |
| male_pituitary_control vs female_pituitary_control | 102094310 | KV877988.1:1694836-1694951 | CE | -0.55042 | 0.989 |
| male_pituitary_control vs female_pituitary_control | 102096025 | KV877995.1:1641113-1645422 | TE | -0.50189 | 0.989 |
| male_pituitary_control vs female_pituitary_control | 102096025 | KV877995.1:1641108-1641112 | TE | 0.49767 | 0.992 |
| male_pituitary_control vs female_pituitary_control | 102088438 | KV877996.1:174269-174359 | AA | -0.31434 | 0.964 |
| male_pituitary_control vs female_pituitary_control | 102095595 | KV877999.1:2526570-2526590 | CE | -0.40246 | 0.97 |
| male_pituitary_control vs female_pituitary_control | 102095850 | KV877999.1:2586503-2586572 | CE | -0.41735 | 0.98 |
| male_pituitary_control vs female_pituitary_control | 102093744 | KV878001.1:1334140-1334178 | CE | -0.52166 | 0.992 |
| male_pituitary_control vs female_pituitary_control | 102094490 | KV878004.1:3082306-3082345 | AD | 0.75659 | 1.0 |
| male_pituitary_control vs female_pituitary_control | 102089575 | KV878006.1:1348283-1348402 | CE | 0.43934 | 0.98 |
| male_pituitary_control vs female_pituitary_control | 102097941 | KV878011.1:14981792-14982023 | CE | -0.44676 | 0.978 |
| male_pituitary_stress vs female_pituitary_stress | 102091116 | CM007525.1:59799905-59799908 | AA | 0.59135 | 0.992 |
| male_pituitary_stress vs female_pituitary_stress | 102096700 | CM007525.1:133006073-133006175 | CE | 0.40633 | 0.968 |
| male_pituitary_stress vs female_pituitary_stress | 102091538 | CM007525.1:67149953-67150062 | AD | 0.34744 | 0.959 |
| male_pituitary_stress vs female_pituitary_stress | 110357300 | CM007525.1:58321648-58321768 | AA | -0.36005 | 0.975 |
| male_pituitary_stress vs female_pituitary_stress | 102084053 | CM007525.1:7496387-7497242 | CE | -0.13223 | 0.95 |
| male_pituitary_stress vs female_pituitary_stress | 102086396 | CM007525.1:85143664-85143935 | CE | -0.359 | 0.961 |
| male_pituitary_stress vs female_pituitary_stress | 102085809 | CM007525.1:134991337-134991402 | CE | -0.3469 | 0.957 |
| male_pituitary_stress vs female_pituitary_stress | 102090315 | CM007525.1:126533740-126533748 | CE | 0.41805 | 0.968 |
| male_pituitary_stress vs female_pituitary_stress | 102093180 | CM007525.1:31143697-31143759 | AA | 0.34946 | 0.959 |
| male_pituitary_stress vs female_pituitary_stress | 106885273 | CM007525.1:69780088-69780147 | CE | -0.37155 | 0.968 |
| male_pituitary_stress vs female_pituitary_stress | 102092269 | CM007525.1:30356089-30356132 | AA | 0.4823 | 0.977 |
| male_pituitary_stress vs female_pituitary_stress | 102084802 | CM007525.1:9550771-9551199 | TE | 0.49226 | 0.991 |
| male_pituitary_stress vs female_pituitary_stress | 102084802 | CM007525.1:9551199-9551199 | TE | -0.50562 | 0.989 |
| male_pituitary_stress vs female_pituitary_stress | 102098594 | CM007535.1:3226930-3226991 | AA | 0.32757 | 0.983 |
| male_pituitary_stress vs female_pituitary_stress | 102097851 | CM007535.1:16385141-16385281 | CE | 0.36869 | 0.955 |
| male_pituitary_stress vs female_pituitary_stress | 102097709 | CM007536.1:15597840-15597864 | AA | 0.4652 | 0.993 |
| male_pituitary_stress vs female_pituitary_stress | 102091392 | CM007536.1:4272039-4272098 | CE | -0.36076 | 0.977 |
| male_pituitary_stress vs female_pituitary_stress | 102091392 | CM007536.1:4272243-4272368 | CE | -0.37067 | 0.979 |
| male_pituitary_stress vs female_pituitary_stress | 102084295 | CM007536.1:7273900-7276113 | AD | -0.42918 | 0.963 |
| male_pituitary_stress vs female_pituitary_stress | 102084155 | CM007537.1:15890076-15890116 | AA | 0.51487 | 0.975 |
| male_pituitary_stress vs female_pituitary_stress | 106891296 | CM007537.1:15909358-15909492 | CE | -0.34546 | 0.975 |
| male_pituitary_stress vs female_pituitary_stress | 102095946 | CM007537.1:12539802-12539911 | AA | 0.33778 | 0.98 |
| male_pituitary_stress vs female_pituitary_stress | 106145760 | CM007538.1:1164182-1164225 | CE | -0.41961 | 0.974 |
| male_pituitary_stress vs female_pituitary_stress | 102086043 | CM007538.1:2849844-2849980 | CE | -0.41749 | 0.993 |
| male_pituitary_stress vs female_pituitary_stress | 105403301 | CM007538.1:6075962-6076043 | CE | -0.50566 | 0.99 |
| male_pituitary_stress vs female_pituitary_stress | 102097435 | CM007539.1:10467972-10468033 | CE | 0.31595 | 0.957 |
| male_pituitary_stress vs female_pituitary_stress | 104022714 | CM007540.1:615289-615433 | CE | -0.13096 | 0.959 |
| male_pituitary_stress vs female_pituitary_stress | 106146053 | CM007540.1:2224917-2224970 | CE | 0.41033 | 0.974 |
| male_pituitary_stress vs female_pituitary_stress | 102086064 | CM007541.1:8335590-8335645 | CE | -0.10434 | 0.957 |
| male_pituitary_stress vs female_pituitary_stress | 102086064 | CM007541.1:8335891-8335983 | CE | -0.1143 | 0.954 |
| male_pituitary_stress vs female_pituitary_stress | 102083544 | CM007542.1:1771983-1772076 | AA | 0.39966 | 0.965 |
| male_pituitary_stress vs female_pituitary_stress | 102096825 | CM007526.1:45369874-45369948 | CE | -0.48967 | 0.998 |
| male_pituitary_stress vs female_pituitary_stress | 102090423 | CM007526.1:89516219-89516383 | CE | -0.12147 | 0.967 |
| male_pituitary_stress vs female_pituitary_stress | 102088013 | CM007526.1:98233032-98233080 | CE | -0.26857 | 0.961 |
| male_pituitary_stress vs female_pituitary_stress | 102090657 | CM007526.1:74424270-74424340 | CE | 0.52613 | 0.964 |
| male_pituitary_stress vs female_pituitary_stress | 110365053 | CM007526.1:134463895-134464045 | CE | -0.53887 | 0.993 |
| male_pituitary_stress vs female_pituitary_stress | 102083569 | CM007526.1:37820348-37820389 | CE | -0.34494 | 0.971 |
| male_pituitary_stress vs female_pituitary_stress | 102084570 | CM007526.1:72552226-72552353 | AA | 0.47235 | 0.991 |
| male_pituitary_stress vs female_pituitary_stress | 102098693 | CM007543.1:7917897-7918037 | CE | -0.14167 | 0.996 |
| male_pituitary_stress vs female_pituitary_stress | 102088856 | CM007543.1:1686166-1686280 | CE | 0.11869 | 0.973 |
| male_pituitary_stress vs female_pituitary_stress | 102088856 | CM007543.1:1686355-1686491 | CE | 0.13918 | 0.963 |
| male_pituitary_stress vs female_pituitary_stress | 102092628 | CM007543.1:4697777-4697789 | AD | -0.35889 | 0.976 |
| male_pituitary_stress vs female_pituitary_stress | 102087669 | CM007543.1:440939-441004 | AD | 0.4116 | 0.955 |
| male_pituitary_stress vs female_pituitary_stress | 102089528 | CM007544.1:1179217-1179442 | CE | -0.13582 | 0.974 |
| male_pituitary_stress vs female_pituitary_stress | 102086868 | CM007547.1:2531808-2532403 | CE | -0.60868 | 0.978 |
| male_pituitary_stress vs female_pituitary_stress | 102086868 | CM007547.1:2532500-2532640 | CE | -0.65627 | 0.979 |
| male_pituitary_stress vs female_pituitary_stress | 102098182 | CM007547.1:144272-144553 | CE | -0.10493 | 0.961 |
| male_pituitary_stress vs female_pituitary_stress | 102092603 | CM007548.1:46244-46351 | CE | -0.13476 | 0.953 |
| male_pituitary_stress vs female_pituitary_stress | 102084175 | CM007550.1:966115-966239 | CE | 0.47245 | 0.989 |
| male_pituitary_stress vs female_pituitary_stress | 102088697 | CM007527.1:44153164-44153366 | CE | -0.47292 | 1.0 |
| male_pituitary_stress vs female_pituitary_stress | 102094122 | CM007527.1:89826613-89826804 | CE | -0.31338 | 0.951 |
| male_pituitary_stress vs female_pituitary_stress | 102096733 | CM007527.1:24222058-24222066 | CE | 0.34012 | 0.999 |
| male_pituitary_stress vs female_pituitary_stress | 102096733 | CM007527.1:24219477-24219509 | CE | -0.53992 | 1.0 |
| male_pituitary_stress vs female_pituitary_stress | 102096733 | CM007527.1:24207014-24207088 | AD | 0.14327 | 0.962 |
| male_pituitary_stress vs female_pituitary_stress | 102087294 | CM007527.1:15416415-15416486 | CE | 0.40744 | 0.961 |
| male_pituitary_stress vs female_pituitary_stress | 102085833 | CM007527.1:1585956-1585956 | TS | 0.51437 | 0.995 |
| male_pituitary_stress vs female_pituitary_stress | 102085833 | CM007527.1:1585293-1585955 | TS | -0.50872 | 0.992 |
| male_pituitary_stress vs female_pituitary_stress | 102086811 | CM007527.1:1411593-1411613 | CE | 0.36553 | 0.969 |
| male_pituitary_stress vs female_pituitary_stress | 102097572 | CM007528.1:37488561-37488680 | CE | -0.51132 | 0.994 |
| male_pituitary_stress vs female_pituitary_stress | 102098275 | CM007528.1:52695294-52695297 | AA | -0.49915 | 0.99 |
| male_pituitary_stress vs female_pituitary_stress | 102089104 | CM007528.1:45157790-45157816 | CE | -0.29619 | 0.96 |
| male_pituitary_stress vs female_pituitary_stress | 102090822 | CM007528.1:62566927-62567011 | AA | -0.49404 | 0.987 |
| male_pituitary_stress vs female_pituitary_stress | 102090682 | CM007528.1:7879209-7879586 | AD | 0.509 | 0.984 |
| male_pituitary_stress vs female_pituitary_stress | 102098203 | CM007523.1:445642-445843 | CE | -0.29649 | 0.999 |
| male_pituitary_stress vs female_pituitary_stress | 102094290 | CM007523.1:11763101-11763160 | CE | 0.3216 | 0.952 |
| male_pituitary_stress vs female_pituitary_stress | 102083833 | CM007529.1:10046231-10046335 | CE | -0.32946 | 0.961 |
| male_pituitary_stress vs female_pituitary_stress | 102085663 | CM007529.1:9299382-9299482 | CE | 0.45908 | 0.952 |
| male_pituitary_stress vs female_pituitary_stress | 102083595 | CM007529.1:19271242-19271251 | CE | 0.79429 | 1.0 |
| male_pituitary_stress vs female_pituitary_stress | 110363815 | CM007530.1:287477-287816 | RI | 0.21582 | 0.951 |
| male_pituitary_stress vs female_pituitary_stress | 102092265 | CM007530.1:17199453-17199575 | CE | -0.4525 | 0.98 |
| male_pituitary_stress vs female_pituitary_stress | 102093135 | CM007531.1:19016610-19016651 | CE | 0.45907 | 0.969 |
| male_pituitary_stress vs female_pituitary_stress | 102085426 | CM007531.1:12830087-12830218 | CE | 0.25505 | 0.955 |
| male_pituitary_stress vs female_pituitary_stress | 102091346 | CM007531.1:32781562-32782303 | RI | 0.40993 | 0.983 |
| male_pituitary_stress vs female_pituitary_stress | 102083465 | CM007531.1:2972581-2972606 | AD | 0.2366 | 0.955 |
| male_pituitary_stress vs female_pituitary_stress | 102083922 | CM007531.1:24074903-24075800 | AA | 0.76681 | 1.0 |
| male_pituitary_stress vs female_pituitary_stress | 102091432 | CM007531.1:23186188-23186239 | CE | 0.35463 | 0.976 |
| male_pituitary_stress vs female_pituitary_stress | 102091432 | CM007531.1:23186780-23186853 | CE | 0.34322 | 0.963 |
| male_pituitary_stress vs female_pituitary_stress | 102093822 | CM007531.1:603916-604108 | CE | -0.33446 | 0.982 |
| male_pituitary_stress vs female_pituitary_stress | 102092453 | CM007531.1:7917285-7917364 | CE | -0.55818 | 0.995 |
| male_pituitary_stress vs female_pituitary_stress | 102093862 | CM007532.1:3039148-3039201 | AA | 0.22317 | 0.956 |
| male_pituitary_stress vs female_pituitary_stress | 102097377 | CM007532.1:13289223-13289288 | AA | -0.45809 | 0.988 |
| male_pituitary_stress vs female_pituitary_stress | 106145801 | CM007532.1:3449104-3449228 | CE | 0.35599 | 0.951 |
| male_pituitary_stress vs female_pituitary_stress | 110357802 | CM007532.1:23656853-23657036 | CE | 0.35371 | 0.962 |
| male_pituitary_stress vs female_pituitary_stress | 102086573 | CM007532.1:8639003-8639047 | AA | -0.37476 | 0.967 |
| male_pituitary_stress vs female_pituitary_stress | 102098898 | CM007533.1:10673387-10673454 | CE | -0.42337 | 0.967 |
| male_pituitary_stress vs female_pituitary_stress | 102085982 | CM007533.1:15326056-15326149 | CE | 0.53378 | 0.992 |
| male_pituitary_stress vs female_pituitary_stress | 102090586 | CM007533.1:8056455-8056558 | CE | -0.51499 | 0.995 |
| male_pituitary_stress vs female_pituitary_stress | 102087407 | CM007533.1:8726809-8726904 | CE | -0.36234 | 0.957 |
| male_pituitary_stress vs female_pituitary_stress | 102087410 | CM007524.1:1013819-1013912 | CE | -0.24677 | 0.968 |
| male_pituitary_stress vs female_pituitary_stress | 102090591 | CM007524.1:24727852-24727901 | CE | -0.45739 | 0.977 |
| male_pituitary_stress vs female_pituitary_stress | 103901317 | KV877992.1:462798-463076 | AA | -0.42371 | 0.971 |
| male_pituitary_stress vs female_pituitary_stress | 105411610 | KV877994.1:1637599-1637668 | CE | -0.54804 | 0.993 |
| male_pituitary_stress vs female_pituitary_stress | 102091925 | KV878001.1:1026659-1026705 | AD | 0.42815 | 0.961 |
| male_pituitary_stress vs female_pituitary_stress | 102093744 | KV878001.1:1334140-1334178 | CE | -0.32162 | 0.984 |
| male_pituitary_stress vs female_pituitary_stress | 102093886 | KV878003.1:845934-846170 | RI | -0.53568 | 0.997 |
| male_pituitary_stress vs female_pituitary_stress | 102084695 | KV878004.1:1057450-1057473 | CE | 0.3563 | 0.961 |
| male_pituitary_stress vs female_pituitary_stress | 102097185 | KV878008.1:1517986-1518011 | AA | 0.31637 | 0.976 |
| male_pituitary_stress vs female_pituitary_stress | 102097185 | KV878008.1:1518012-1518726 | CE | 0.29127 | 0.95 |
| male_pituitary_stress vs female_pituitary_stress | 102085500 | KV878011.1:7175866-7175940 | CE | -0.38292 | 0.975 |
| male_pituitary_stress vs female_pituitary_stress | 102085500 | KV878011.1:7173974-7174066 | CE | -0.34585 | 0.956 |
| male_pituitary_stress vs female_pituitary_stress | 102096491 | KV878011.1:1431221-1431283 | AA | 0.41293 | 0.993 |
| male_pituitary_stress vs female_pituitary_stress | 102086217 | KV878015.1:1316559-1316615 | AA | 0.35323 | 0.972 |

**
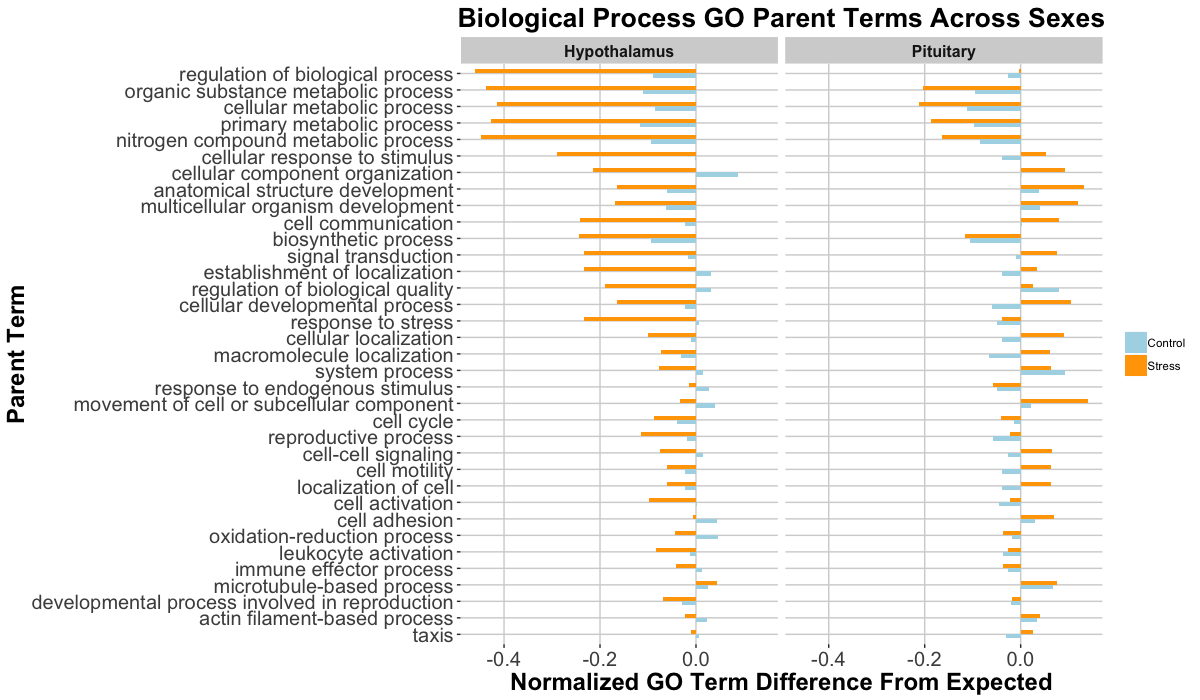
**

Figure S1. Male vs. Female Biological Process GO Analysis


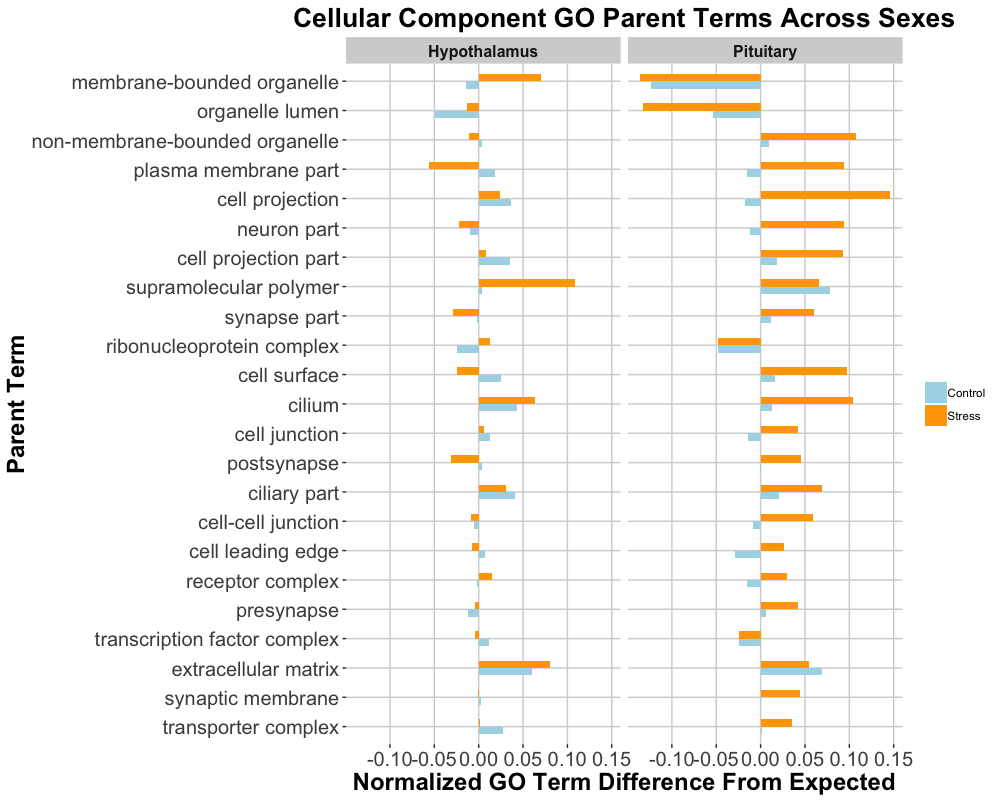


Figure S2. Male vs. Female Cellular Component GO Analysis


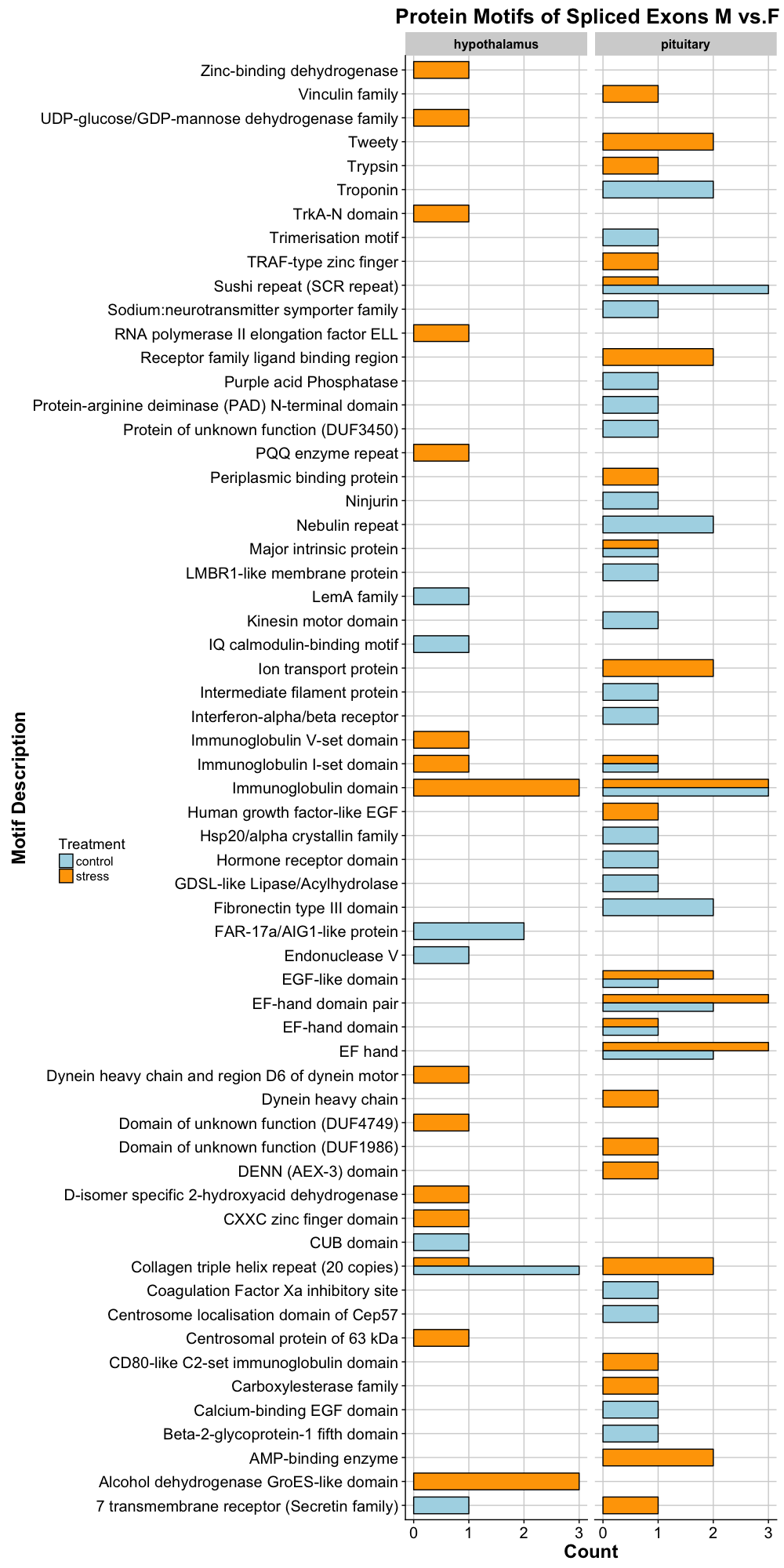


Figure S3. Male vs. Female Exon Motifs


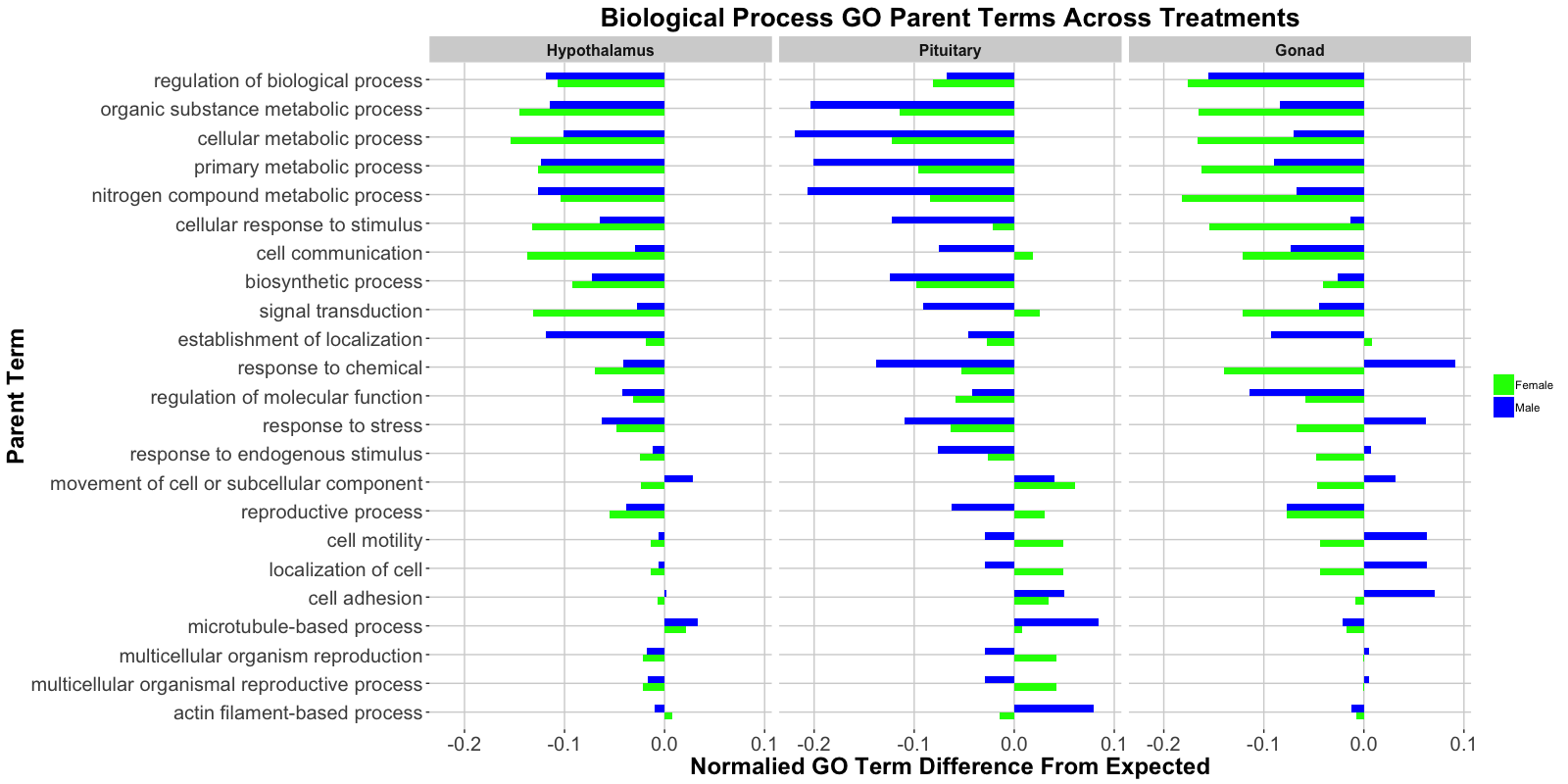


Figure S4. Control vs. Stress Biological Process GO Analysis


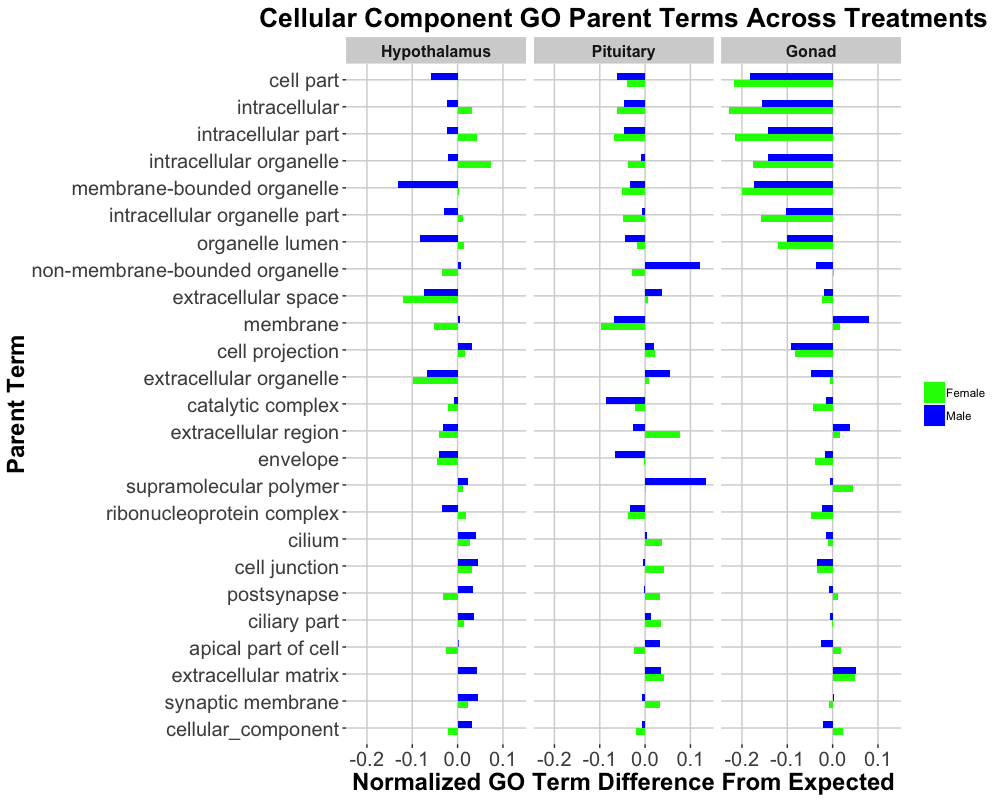


Figure S5. Control vs. Stress Cellular Component GO Analysis


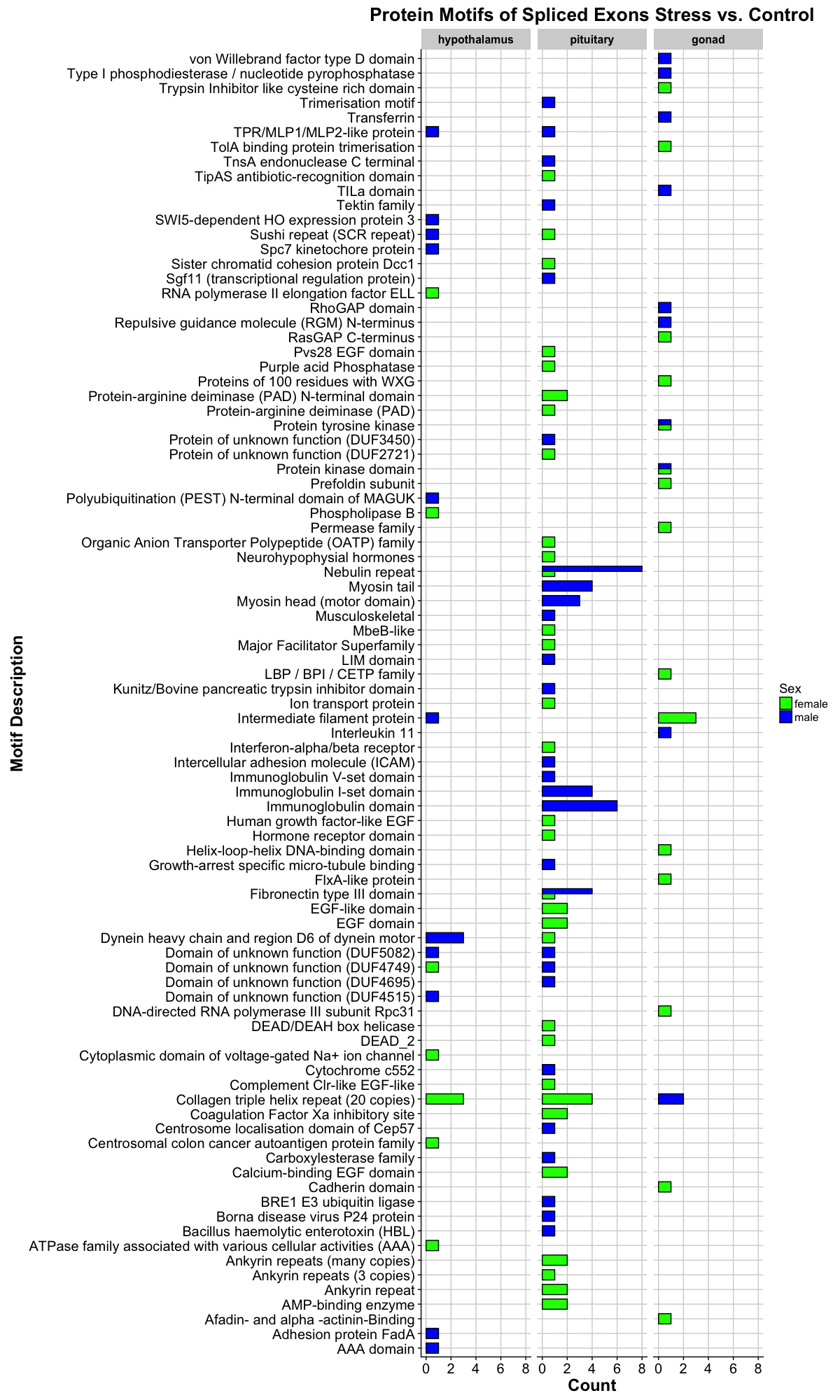


Figure S6. Control vs. Stress Exon Motifs


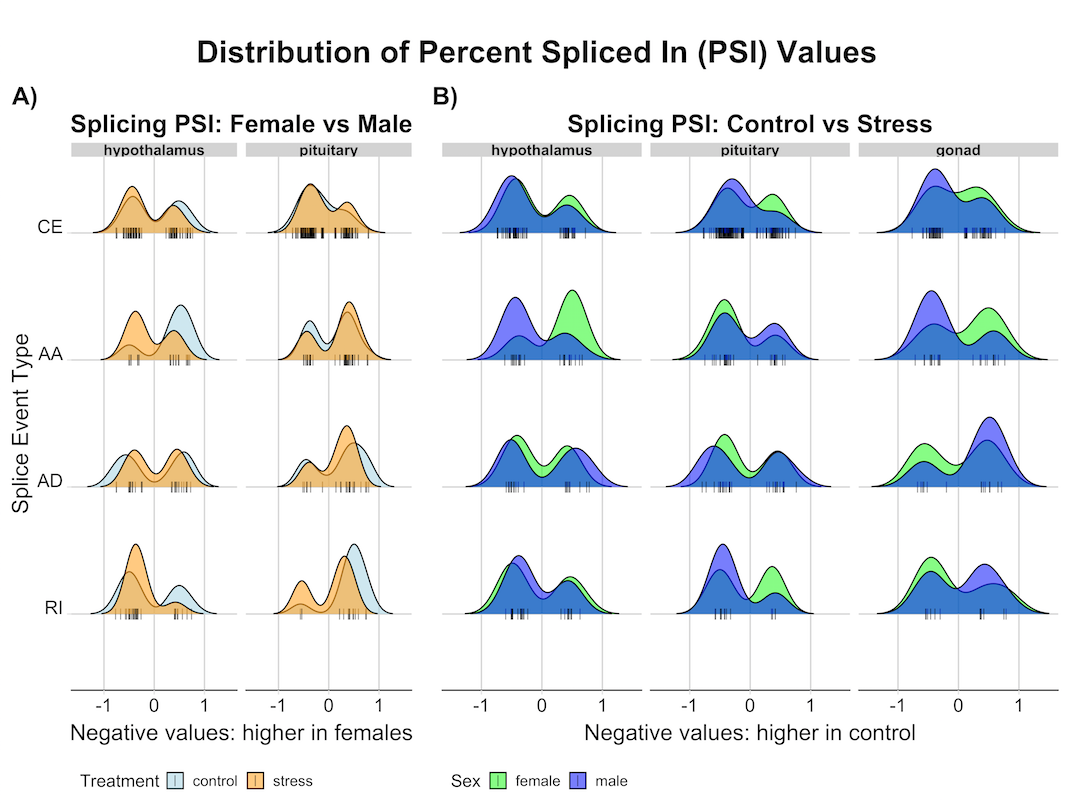


Figure S7. Distribution of Percent Spliced In (PSI) Values for the a) control vs stress analysis and also the b) male vs female analysis. The figure is divided into vertical panels, each representing the tissues labeled a the top The right two panels show the distribution of Percent Spliced In (PSI) values, with “|” symbols at the base of each ridge plot each corresponding to the PSI value of a single event. The heights of the ridge plots should not be compared from one event to another, but within an event between treatments. Splicing event types are displayed along the y-axis (CE: core exon, AA: alternate acceptor, AD: alternate donor, RI: Retained intron). The coloring scheme is the same as previous figures (light blue: control, orange: restraint stress, green: female, blue: male).
